# The life history theory of the Lord of the Rings: a randomized controlled trial of using fact versus fiction to teach life history theory

**DOI:** 10.1186/s12052-022-00160-8

**Published:** 2022-02-16

**Authors:** Carlo C. Maley, Sareh Seyedi

**Affiliations:** Arizona Cancer Evolution Center, Biodesign Institute and School of Life Sciences, Arizona State University, Tempe, USA

**Keywords:** Life history theory, Sexual selection, Kin selection, Sexual dimorphism, Species definition, Tolkien, Lord of the Rings

## Abstract

Does asking students to apply concepts from evolution to a fictional context, compared to a novel biological context, improve their understanding, exam performance or enjoyment of the material? Or does it harm their education by taking time away from true biology? At our institution, we sometimes ask students to apply life history theory to species from fictional movies, television shows or books. Previously, we had used a factual article on life history theory, to supplement our textbook. We wrote an alternative introduction to life history theory (included in the additional files for educational use), using Tolkien’s fictional species from his Lord of the Rings books. We also introduce the biological species definition, sexual selection, sexual dimorphism, kin selection, and the handicap principle, as those concepts arose naturally in the discussion of the fictional species. Life history theory predicts strong correlations between traits affecting reproduction, growth and survival, which are all shaped by the ecology of the species. Thus, we can teach life history theory by asking students to infer traits and aspects of the ecology of a fictional species that have never been described, based on the partial information included in the fictional sources. In a large, third year undergraduate evolution course at Arizona State University, we randomized 16 tutorial sections of a total of 264 students to either read our article on the life history theory of Lord of the Rings, or the factual article we had used previously in the course. We found that the exam performance on life history questions for the two groups were almost identical, except that fans of The Lord of the Rings who had read our article did better on the exam. Enjoyment, engagement and interest in life history theory was approximately a full point higher on a 5-point Likert scale for the students that had read the fictional article, and was highly statistically significantly different (T-test *p* < 0.001 for all questions). There was no difference between the two groups in their familiarity or enjoyment of The Lord of the Rings stories themselves. Reading the article that taught life history theory by applying it to the species of The Lord of the Rings neither helped nor harmed exam performance, but did significantly improve student enjoyment, engagement and interest in life history theory, and even improved exam scores in students who liked The Lord of the Rings. Using fiction to teach science may also help to engage non-traditional students, such as world-builders, outside of our institutions of education. By encouraging students to apply the scientific ideas to their favorite stories from their own cultures, we may be able to improve both inclusivity and education.

**Supplementary Information:**

The online version contains supplementary material available at 10.1186/s12052-022-00160-8.

## Introduction: science fantasy for science

One of the key skills we hope to build in our students is the ability to generalize principles to a novel context. This is central to the process of science, and one of the key drivers of innovation and interdisciplinary research. Such generalization demonstrates a deep understanding of the principle, rather than a simple recall of the examples the student has previously seen. In order to challenge students to generalize concepts to new contexts, teachers can borrow examples from nature that have not been previously covered in class. However, it can also be a powerful motivator and inspiration to apply ideas to examples from fantasy and science fiction that have already shown success in firing students’ imaginations (Bixler 2007), and to utilize the narratives of those stories to enhance education (Vrasidas et al. 2015). The Lord of the Rings has been used previously as an analogy to teach the human immune response (Souza-Hart 2011). Here, we apply this technique to teach life history theory using the creatures of J.R.R. Tolkien’s Middle-Earth.

But first we must acknowledge that Tolkien was not perfect. He was a wonderful philologist and storyteller, but he was not a biologist (Tolkien 1981, #153). One of his express goals in his work was to provide Britain with its own mythology (Carpenter 1977). Thus, we must take care to separate his keen observations from his myth making. This is most clear in his observations about elves. He claims that they are immortal (Tolkien 1955, Appendix A), which is biologically implausible. We may safely dismiss this as mythologizing and the exaggeration of an extremely long lifespan.

Though Tolkien left behind many pages of notes on Middle-Earth, there is only so much that a single observer can catalog. We owe a debt to his son Christopher for collating, editing and publishing those notes that provide our only knowledge of Middle-Earth (Tolkien et al. 1977; Tolkien and Tolkien 1996, 2010; Tolkien 2000). For the rest, for the gaps in Tolkien’s observations, we may apply life history theory to infer and predict many undocumented aspects of the hominids (elves, orcs, dwarves and hobbits) of Middle-Earth.

The life history traits of a species are those characteristics that determine the organism’s likelihood of survival to a given age, and the number of offspring it typically produces at each age. These include traits like lifespan, growth rate, age of sexual maturity, frequency of reproduction, number of infants per birth (e.g., twins), susceptibility to infection, body size, and traits that affect the ability to compete for mates and resources. These traits are shaped by the selective pressures of the ecology of a species, which often leads to correlations between different life history traits. For example, large organisms often live longer, delay reproduction and have fewer offspring than small organisms.

## Hominids of Middle-Earth

The hominids of Middle-Earth show extreme variation for many life history traits. What is known about the hominids of Middle-Earth and what can we infer from that?

### Elves

**Fig. 1:**
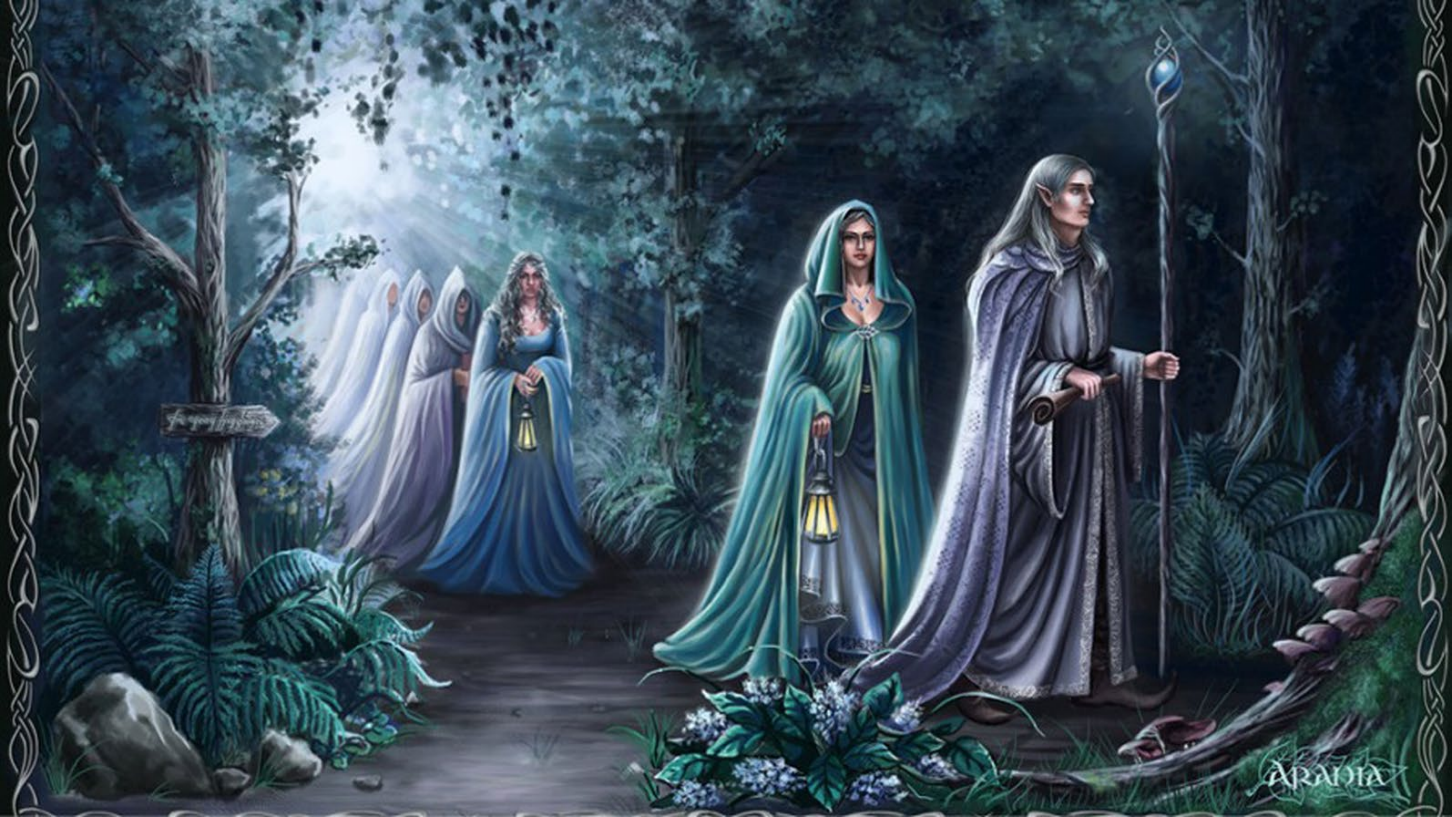
An artist’s rendition of elves (Image by Araniart/CC BY (https://creativecommons.org/licenses/by/3.0)

The elves of Middle-Earth (Fig. 1), are tall, almost ethereal creatures of extremely long lifespans. Though they can walk, talk and dance by 1 year of age, they take 50–100 years to fully mature (Tolkien 2010a). Elves have few children, and long interbirth intervals (Tolkien 2010a). Tolkien states that they are immortal (Tolkien 1955, Appendix A; 2010a), in that their faculties do not decline with age (i.e., senesce) (Tolkien 1981, #153), but we may take this as an exaggeration of an incredibly long lifespan. Though they can be killed by violence, they have better wound healing abilities, stamina and resistance to disease compared to humans (Tolkien 2010b, Note 5). The combination of long life, disease resistance and effective wound healing should not be surprising. If there has been natural selection on elves to evolve extremely long lifespans, the same ecological conditions would select for the prevention of premature death from wounds or disease.

What are those ecological conditions that select for a long lifespan? If there is very little risk of death from predators, competitors or diseases, called “external sources of mortality”, then populations will grow until they become limited by resources. At that point, selection shifts from external sources of mortality to competition for limited resources. Competition for limited resources usually selects for the evolution of what are called slow life history strategies. These include a constellation of traits including long lifespans, large body size, high levels of investment in maintaining those bodies, few offspring, long periods of development before reaching sexual maturity, and high levels of parental investment in those offspring. Elves’ lifespan, height, slow growth to maturity, and superior wound healing and disease resistance are all consistent with elves exhibiting the slowest life history strategy known in mammals. It may only be rivaled by some trees on Earth, such as bristlecone pine trees, that can live thousands of years (Lanner and Connor 2001).

What does all of this imply about elves that was not documented by Tolkien? Long interbirth intervals and few children implies that there must be very high levels of parental investment in their elflings. Matings and births must be very important events in elven culture. Long lifespan is also strongly selected in species for which older individuals are better able to compete for mates and status, and otherwise successfully reproduce. This suggests that older elves likely have higher status and more successful matings than younger elves. We might question Tolkien’s claim that elves tend to monogamously marry and produce offspring shortly after attaining sexual maturity, losing interest in sex thereafter (Tolkien 2010a).

Elves’ extremely long lifespans suggest that they were able to effectively avoid death from conflict with the other hominids of Middle-Earth, as well as other elves, during most of their evolution. They do appear to live in extremely well guarded enclaves in Middle-Earth (Tolkien 1967). This also implies that they must expend a great deal of their resources and time on limiting risks to themselves. This may help to explain their insular nature, their high levels of competence at various survival skills, and their preference for use of bow and arrow over more risky forms of close combat. Their long lifespans and investment in somatic maintenance suggests that, like elephants, they probably also have effective mechanisms for dealing with DNA damage and preventing cancer (Abegglen et al. 2015).

Elves’ very long lifespans and slow reproduction calls into question why they would ever risk leaving their safe enclaves. One possibility is that there has been kin selection for sending out scouts to identify potential threats. The risk of scouting to the individual scout might be outweighed by the survival benefit to its relatives by identifying and defusing external threats, thereby preserving and spreading the alleles of genes in the relatives that made the scouts willing to take that risk. Another possibility is that leaving the enclave is a potential high risk/high payoff strategy for acquiring resources or prestige which would likely translate into increased reproductive opportunities. Given their long evolutionary history where competition for resources was probably the primary selective pressure, it would not be surprising if there has been sexual selection on elves to choose mates that have proven effective at acquiring resources. In contrast, there is evidence that there is little competition between males or between females (intra-sexual competition). Such intra-sexual competition often leads to the evolution of sexual dimorphism (physical differences between the sexes of a species), as seen in the large antlers male moose, but not female moose, grow every year. However, elves have even less sexual dimorphism than humans (Tolkien 2010a), who themselves have less sexual dimorphism than most primates (Plavcan 2012; Frayer and Wolpoff 1985). The lack of sexual dimorphism suggests that there is little reproductive skew in elves. That is, even high status elves probably do not monopolize multiple mates. This is supported by Tolkien’s observation that they are highly monogamous (Tolkien 2010a).

### Orcs (a.k.a., Goblins, Uruk)

**Fig. 2:**
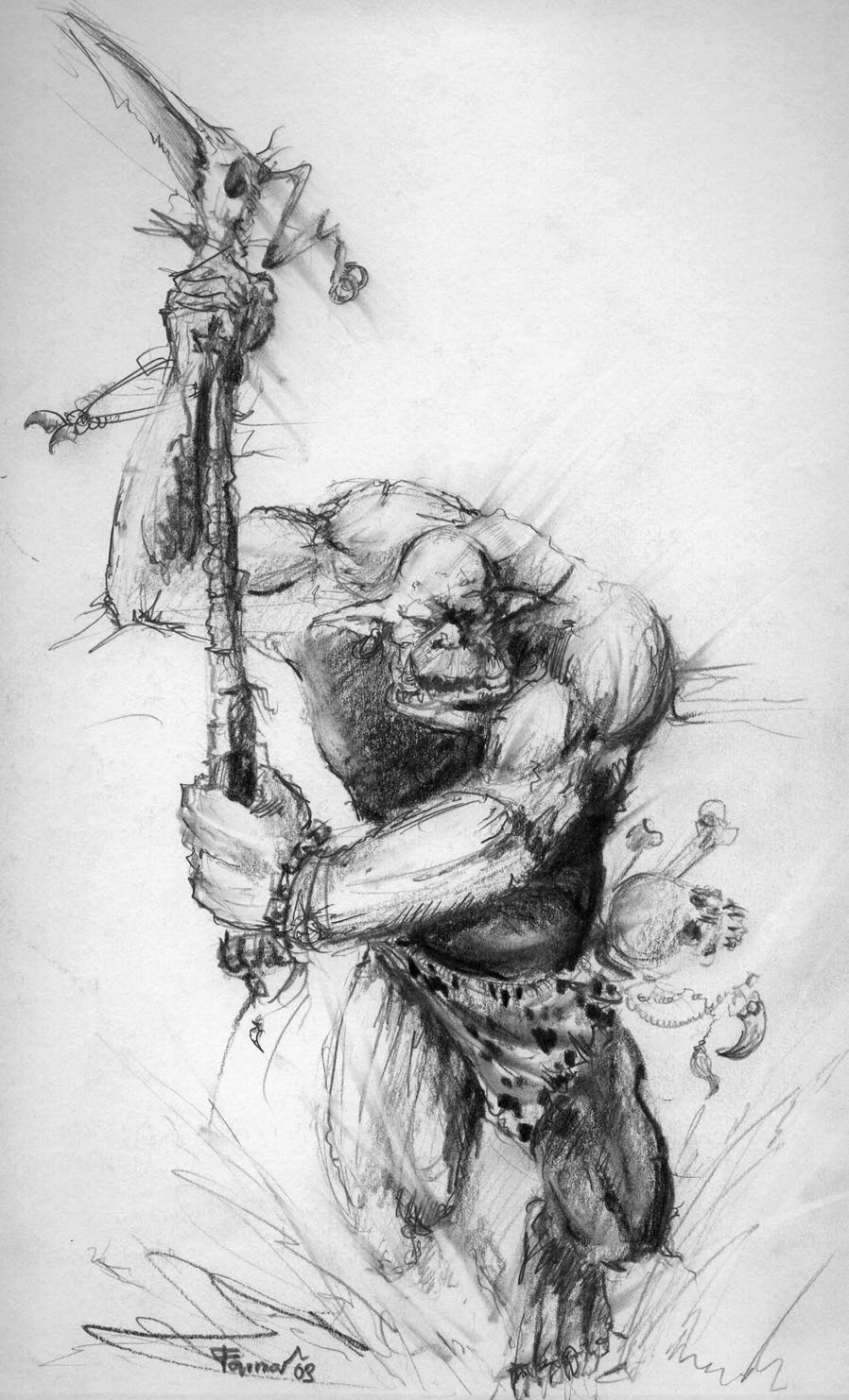
An artist’s image of an orc. Given the small size of most orcs, the skull on its belt probably belonged to another orc or a hobbit. Alternatively, this might be a reproduction of an Uruk-hai, one of the larger orcs (Image by farmerownia (www.farmerownia.pl) ( BY-SA ())

The orcs of Middle-Earth (Fig. 2) are small (Tolkien 1955, Appendix A, 1971), so small in fact that 3-foot (1 m) tall hobbits in disguise can pass for orcs (Tolkien 1955, The Land of Shadow). They are generally squat and broad (Tolkien 1981, #210), mostly live underground and are sensitive to sunlight (Tolkien 1971). Orcs have short lifespans and rapidly multiply in their enclaves (Tolkien et al. 2010; Tolkien 1996a). In short, they have evolved a fast life history strategy. And it is no wonder, for in contrast to elves, there is clearly a great deal of external mortality for orcs. They are not only hunted down and killed by the other hominids of Middle-Earth, but there is intense violence between orcs and conflict over status that is often lethal (Tolkien 1955, 1971). Any species that experiences such high levels of mortality is under selection to reproduce quickly before they are killed. This means that they must reach sexual maturity quickly, which generally selects for a smaller body size, as does living underground.

The violent clashes between orcs over hierarchy suggest that orcs probably have extreme reproductive skew, with high status males monopolizing mating opportunities. It is unclear if there is also mate competition among female orcs. Unfortunately, Tolkien did not observe any female orcs, and so we know little about them. If they are like other fast life history mammals, high mortality rates would have led to the evolution of more than two mammary glands in female orcs, large numbers of offspring, probably with multiple infants per birth. In the extreme, selection for fast life histories can lead to semelparity—organisms that reproduce only once, but massively, in their life and then die. It is unclear if orcs are semelparous, but it seems more likely that they would reproduce like other fast life history mammals, such as mice, that repeatedly have large litters. Selection for fast life history strategies also selects for low levels of parental investment. This may have led to the evolution of low levels of oxytocin and affiliative bonding between orcs, which would be consistent with descriptions of their interactions (Tolkien 1955, 1971).

Boom and bust cycles, and other causes of large population fluctuations, also select for fast life histories. If there is a sudden expansion of resources, or the population is knocked down well below the carrying capacity of the environment, then individuals who can reproduce the fastest will fill that vacuum and dominate future generations. We know that a number of wars caused large population fluctuations in orcs. In fact, the War of Wrath that ended the first age almost led to their extinction (Tolkien et al. 1977). Such significant population bottlenecks tend to lead to high frequencies of genetic diseases.

If an orc survived to old age, it probably would die of cancer. In species that suffer a high degree of external mortality and have little parental investment, there is no selection for maintaining the body late in life, including cancer suppression. They also likely suffered from high levels of infectious disease, for the same reason. Fast life history organisms that devote the majority of their resources to rapid growth and reproduction will out-compete those that devote their resources to a strong immune system and cancer suppression, only to be killed through violence. The one exception might be wound healing. Given the levels of violence in the experience of orcs, they were probably selected for effective wound healing.

One common feature of fast life history organisms is that they tend to disperse widely, like weeds that persist by quickly colonizing new disturbances in the environment. Small body size and low investment in their bodies might render orcs at a disadvantage when directly competing with other hominids of Middle-Earth, which is supported by the fact that they lost most of the documented wars with the other hominids over the ages. So instead of directly competing, there would have been selection on orcs to tend to migrate in order to find environments where they could thrive without direct contact with humans, elves or dwarves.

Finally, we should note that orcs are clearly not the unthinking brutes that they were often portrayed as. The fear and long standing deadly conflict between orcs and the other hominids of Middle-Earth probably biased reports of their nature. The size and organization of their armies, including forged weapons and armor (regardless of their poor quality), as well as complex machinery of war (e.g., catapults) and mining (including engines and explosives) (Tolkien 1982), speaks to a highly developed civilization and industry.

### Dwarves

**Fig. 3:**
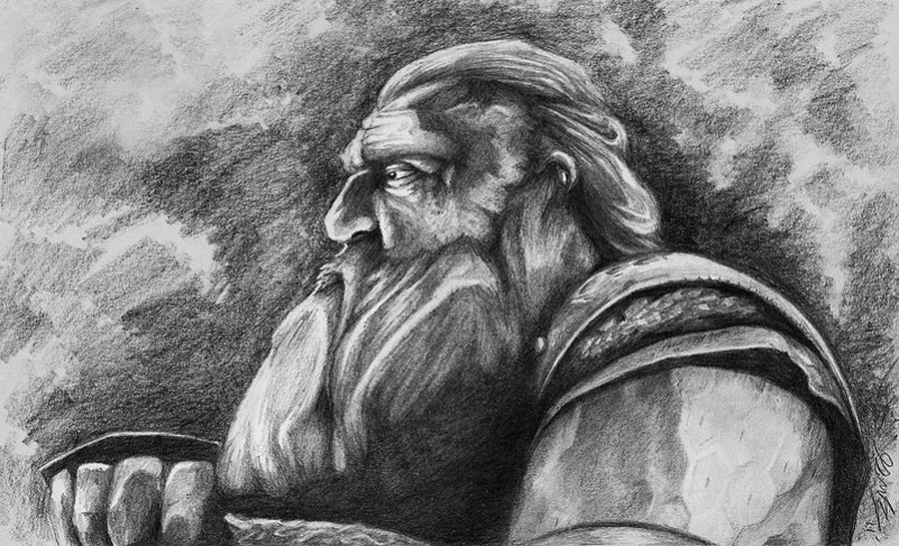
An artist’s drawing of a dwarf, probably male (Image by Perrie Nicholas Smith/CC BY-SA (https://creativecommons.org/licenses/by-sa/4.0)

Dwarves are the mortal enemies of orcs, and very different from them biologically (Fig. 3). Dwarves have very long lifespans, averaging around 250 years, with very little senescence until the last decade of their life (Tolkien 1996b). They take 30 years to grow to adulthood and do not usually reproduce before 90 years of age (Tolkien 1955, Appendix A). In other words, they have a much slower life history strategy than orcs or even humans. They are short and generally live underground, though they eat the same kind of foods as humans and hobbits (Tolkien 1982, 1996c). They are resistant to most diseases, except obesity (Tolkien 1996b), and are even resistant to damage from fire (Tolkien et al. 1977). Dwarves can withstand suffering and toil more than the other hominids of Middle-Earth (Tolkien et al. 1977). This all adds up to a clear picture of a slow life history strategy for dwarves. This is pretty typical for creatures that live underground (Novikov and Burda 2013), when living underground provides a good defense against predators and other sources of violent death. However, life history strategies can be flexible and respond to signals from the environment. Given the well documented population fluctuations of dwarves, due to wars (Tolkien 1955, Appendix A; Tolkien et al. 1977) and dragon attacks (Tolkien 1982), it is quite likely that dwarves can shift into a faster life history strategy to exploit the newly available space and resources after such a population bottleneck.

Dwarves do not generally farm for themselves and there are no mentions of them being hunter-gatherers (Tolkien et al. 1977). Because Dwarves live underground but depend on food grown above ground, they must form symbiotic relationships with other hominids to supply their food. Indeed there are multiple reports of such relationships with humans (Tolkien 1955, Appendix A), elves (Tolkien et al. 1977) and even hobbits (Tolkien 1996c). Those relationships go beyond trade for food. Dwarves are expert miners and smiths who provide high quality arms and armor to both elves and humans who then fight off their mutual enemies (Tolkien et al. 1977). Although there is little mention of it, we might expect dwarves to protect their suppliers of food and allies in the dwarven strongholds during times of strife.

Dwarves are unusual in that there are twice as many males as females (Tolkien 1955, Appendix A). This sex bias is thought to slow their population growth rate (Tolkien 1955, Appendix A). Normally, such a skewed sex bias would lead to intense competition among the males for mating opportunities, and that intra-sexual competition often leads to sexual dimorphism. However, dwarves are notorious for their lack of sexual dimorphism. In fact, female dwarves look so much like male dwarves, including sporting beards, that they are often mistaken for males (Tolkien 1955, Appendix A). Given the sex bias, one might expect dwarves to be polyandrous, with females taking multiple male mates, but this does not appear to be the case. Dwarves are generally monogamous, taking only one husband or wife in their lives, and are jealous of both wealth and their mates (Tolkien 1955, Appendix A). Instead of male–male competition or polyandry, many male dwarves opt out of competing for mates and prefer to focus on their craft. This is reminiscent of many species in which relatives forgo their own reproduction to help with the care and survival of their close kin, sometimes called “helpers in the nest” (Komdeur 2010). Since their kin also carry the alleles for such non-reproductive helping behavior, those alleles can spread in the population. This is a phenomenon called kin selection.

The dwarves’ obsession with treasure, and particularly beards, suggests that sexual selection among dwarves focuses on those traits. In fact, dwarven beards come in a variety of colors, from yellow, to blue, to white, like the colorful plumage of birds that appears to have evolved through sexual selection (Safran and McGraw 2004). Luxuriant beards may also help to advertise a low disease burden and thus a desirable mate, an idea called the handicap principle (Johnstone 1995; Zahavi 1975). Similarly, sexual selection for mates with abundant resources would explain dwarves’ lust for gold and their motivation to leave their safe underground dwellings to seek treasure (Tolkien 1982). The focus on treasure is likely also explained by dwarves’ dependency on trade with other hominids to supply them with food and other necessities that they cannot procure themselves. It is no wonder that they have invested so much effort and skill in mining for precious metals and gems, as well as crafting highly desired tools and artefacts from them.

### Hobbits

**Fig. 4:**
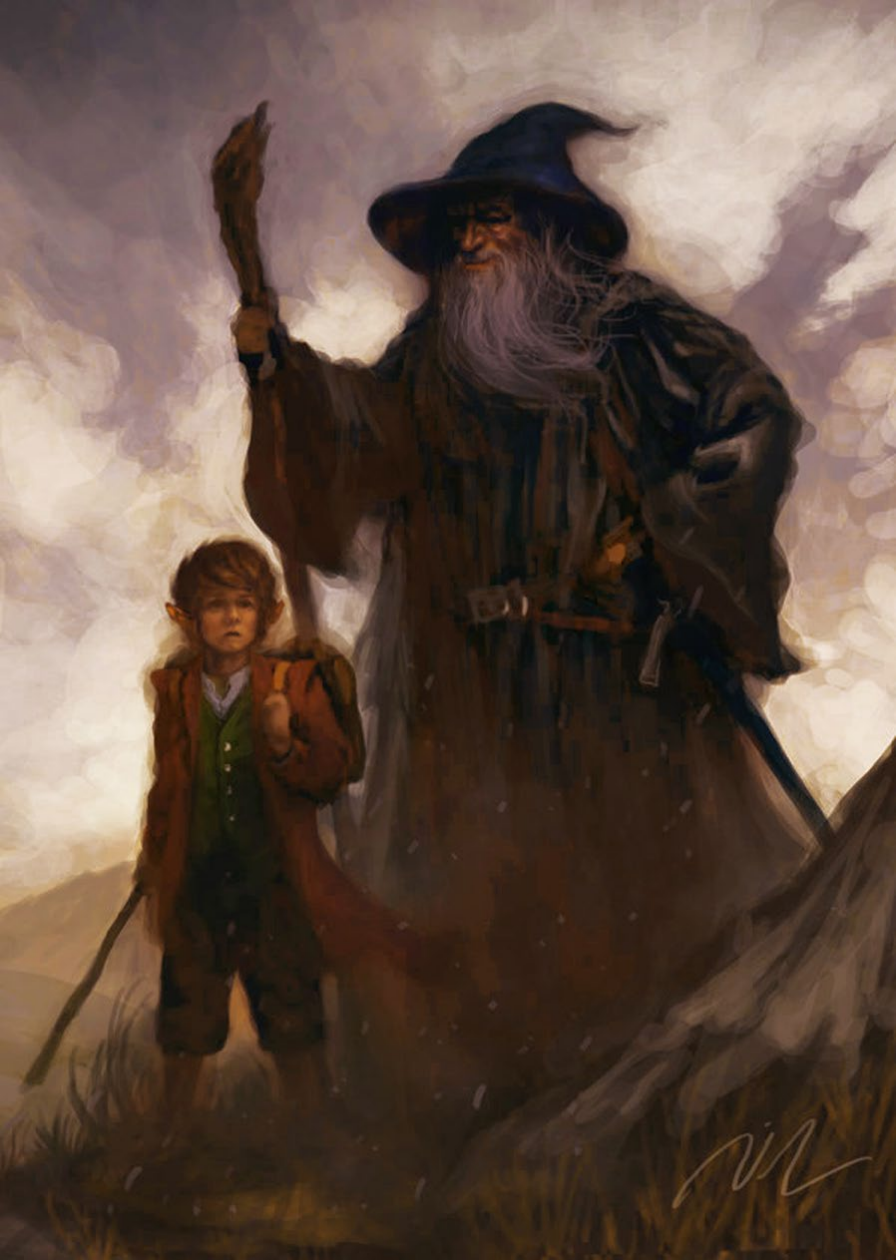
An artist’s rendition of a hobbit. Shown here with a human sized wizard, for scale (Image by Joel Lee (maxbat)/CC BY-SA (https://creativecommons.org/licenses/by-sa/4.0)

Hobbits, called halflings by humans, are short, usually between 2 and 4 feet tall (around 1 m) (Tolkien 1967, Prologue) (Fig. 4). They traditionally live underground in cozy dwellings of large, multi-generational families, though some now live above ground in houses that maintain some of the architecture of a hobbit hole. They have large families and live a little longer than humans, averaging approximately 100 years (Tolkien 1967). They are exceptionally fond of food and drink. They typically consume six meals a day (seven if you count “second breakfast”) (Tolkien 1967, Prologue). The fact that they consume so much food may well limit the carrying capacity of their environment and explain in part their relatively limited population size, compared to humans.

They are shy of humans and other “big folk,” and are excellent at hiding from them (Tolkien 1967, Prologue). They are neither prone to war nor violent strife among themselves (Tolkien 1967, Prologue). They seem to be protected from the violence of the other hominids by the service of the human rangers and at least one wizard (Tolkien 1967), suggesting that they are good at coopting the investment of other hominids of Middle-Earth. They also have strong social norms that encourage proper behavior and discourage risk taking as well as travel. Most of this speaks of a slow life history strategy, though not as slow as dwarves or elves. This is consistent with few sources of external mortality for hobbits. For any slow life history species, the cost of risks is higher than for fast life history species, as the slow life history organisms must generally survive for a long time to attain resources and mates, as well as rear their young. This may explain hobbits’ aversion to risk.

Unlike most slow life history species, hobbits do have many children and only grow to a small size. However, their multigenerational families suggest significant parental investment in their young. Their small size probably evolved in part as an adaptation to underground living rather than adult mortality, though they clearly spend a fair amount of time farming above ground. Their slow life history traits suggest that hobbits probably have excellent defenses against both infectious disease and cancer.

Are humans, elves, ocrcs, dwarves, and hobbits different species, or just different variations within a single species? There is evidence for both alternatives (Box 1).

Box 1: Are the hominids of Middle-Earth different species? With such different life history strategies, we might ask if the different hominids of Middle-Earth are different species. Biologists generally define a species as a reproductively isolated population (Queiroz 2007). That is, two populations are different species if they cannot interbreed, producing fertile offspring. Clearly, elves and humans may successfully interbreed, producing fertile half-elves in at least three documented unions (Idril and Tuor; Luthien and Beren; Arwen^1^ and Aragorn) (Tolkien 1955, Appendix A). Elrond the half-elven was the grandson of both Idril and Tuor as well as Luthien and Beren. Elrond himself is fertile, having produced two sons, Elladan and Elrohir, as well as a daughter, Arwen, who in turn was able to successfully breed with a human, Aragorn, son of Arathorn. So humans and elves appear to be different morphs of the same species with extremely divergent life history strategies. Tolkien supports this view in a letter stating that “Elves and Men are just different aspects of the Humane.” (Tolkien 1981, #181).

Orcs can also interbreed with humans (Tolkien et al. 2010). There are suggestions of admixture of human genes into the fighting Uruk-hai that Saruman bred, making them larger than normal orcs and allowing them to operate comfortably in daylight (Tolkien et al. 2010). Saruman’s armies also included half-orcs (Tolkien 1971). In addition, there are references to half-orcs that Saruman bred and used as spies (Tolkien 1967) and guards (Tolkien 1955, Appendix A). Legend has it that orcs were either derived from elves (Tolkien et al. 1977) or from humans (Tolkien et al. 2010), and so it is likely that orcs, humans and elves are all part of a single species, spanning extreme ranges of life history strategies.

Despite hobbits being more closely related to humans than humans are to elves (Tolkien 1967, Prologue), there is no mention of successful matings between hobbits and humans, or between hobbits and any of the other hominids of Middle-Earth. Similarly, there is no mention of successful matings between dwarves and other hominids, suggesting true species barriers between them and the other hominids of Middle-Earth. In fact, hobbits may be in the process of dividing into three species as the three hobbit breeds (Harfoots, Stoors and Fallohides) have evolved different physical characteristics, which suggests limited interbreeding between the hobbit breeds. This reproductive isolation was probably reinforced by their preferences for different habitats: Harfoots in the highlands and hillsides, Stoors in the flatlands and riversides, and Fallohides in forests (Tolkien 1967, Prologue).

Colin’s predicted relationships between Tolkien’s hominids using a character based phylogenetic reconstruction method (Colin et al. 2021). His phylogeny groups orcs and elves together (due to their pointed ears and loss of facial hair), suggesting that they are more closely related to each other than they are to humans. Though his analysis suggests that Dwarves and hobbits are more closely related to humans than they are to elves and orcs, the evidence of reproductive barriers does not support this.

### The lessons of life history theory and evolutionary ecology

**Fig. 5:**
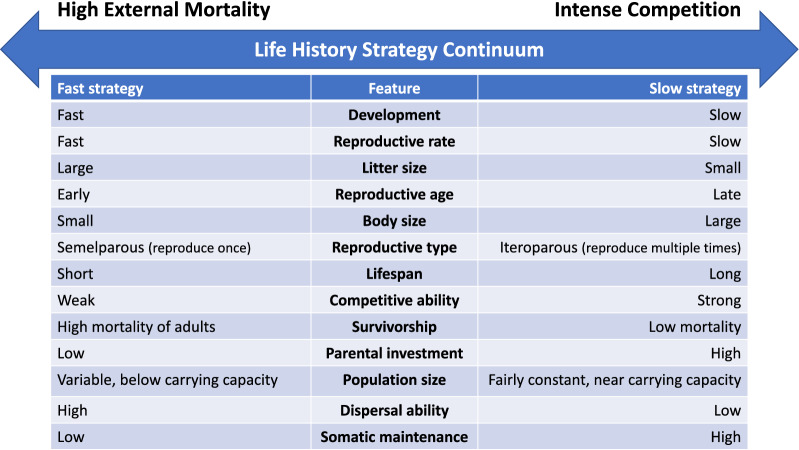
Life history strategy continuum. Ecologists often put life history strategies on a continuum from fast to slow. Selection due to high levels of external mortality (like predation) select for fast life history strategies while low levels of external mortality but high levels of competition within a species tend to select for slow life history strategies

The ecology of a species shapes its life history traits, through natural selection. Many of those traits are correlated and are often described as forming a continuum from fast to slow life history strategies (Fig. 5). It is not the case that one life history strategy is better than another, per se. Which life history strategy works best depends on the environment and the other species in that environment. If members of a species are rapidly and often killed off, perhaps by predators, or maybe environmental disturbances like fires and droughts, then those individuals that can reach sexual maturity quickly and reproduce rapidly, producing large litters, will tend to leave behind more offspring than the individuals that grow more slowly, investing in a robust body only to be killed before they can reproduce.

In contrast, if there is very little external mortality, then a population will tend to grow until it reaches some limitation of resources. At that point, the organisms that can most effectively compete for those limiting resources will tend to survive and reproduce better than their ineffective competitors. These ecologies select for organisms that can build robust bodies and can live a long time (Fig. 5), fending off diseases. They also select for organisms that invest a lot of resources in their young, protecting them and raising them until they can effectively compete with other members of their species. Because resources are limited, this generally means that organisms with such slow life history strategies grow slowly and have few offspring. There are a variety of ways that a species might evolve to avoid predation including growing large (Sinclair et al. 2003), living underground (Novikov and Burda 2013), or evolving flight (Wright et al. 2016). This helps to explain why bats have evolved to live so much longer [e.g. 40 years for Brandt’s bat (Munshi-South and Wilkinson 2010)] than other mammals of the same size. Even within the same environment, there can be different niches where different life history strategies can thrive. For example, fast life history weeds and rodents often coexist in forests with slow life history trees, bats, and birds. In addition, many organisms can shift their life history strategies, and even switch between sexual and asexual reproduction, in response to signals from their environment (Fraser and Gilliam 1992; Fournier et al. 2016).

Sexual selection can also have strong effects on the evolution of species. This includes one sex choosing which individuals of the other sex to mate with (inter-sexual competition) as well as members of one sex competing with each other for mating opportunities (intra-sexual competition). If there is a lot of reproductive skew, if a minority of individuals in a population get all the mating opportunities, then competition to be in that reproductive minority can be intense, and selection for traits that provide for success in such competition can swamp other selective pressures [like cancer suppression (Boddy et al. 2015)].

### Experimental evaluation

Are generalizations of biological principles to fictional contexts more effective methods for teaching biology than traditional summaries of biology? To answer this question we randomized 16 tutorial sections of an undergraduate evolution course at Arizona State University to either the “fiction” condition or the “fact” condition. In the fiction condition, students read an earlier draft of the above article (excluding this section, provided as Additional file 1: Text S1), and filled out the fiction worksheet (see Additional file 2: Text S2). In the fact condition, students read Fabien and Flatt 2012, which we had used as supplemental reading for the course in a previous year. They then filled out the Fabien and Flatt factual worksheet (see Additional file 3: Text S3) which only differed from the fiction condition by the example species used. As our course is a 300-level course, required for biology majors, most students in the course are 3rd year biology majors. Each tutorial consisted of up to 19 students. There were four teaching assistants for the course, each of which ran four of the tutorials. The tutorial sessions were randomized to the fact or fiction conditions such that each teaching assistant ran two tutorial sessions of each condition, so that we could control for the different tutors (graduate student teaching assistants). The primary outcomes were performance on the life history section of an exam (for which all students received the same questions, regardless of which article and worksheet they had done) and a survey of student experience, asking the students to score the extent to which they agreed or disagreed with the following statements using a 5-point Likert scale (strongly disagree, disagree, neither agree nor disagree, agree, or strongly agree):

1. The article was fun.
2. The article was clear and understandable.
3. The article was engaging.
4. The article was memorable.
5. The article was helpful in learning life history theory.
6. The article helped me answer the life history questions on the exam.
7. The article made me interested in the topic of life history.
8. I am familiar with Lord of the Rings stories.
9. I like the Lord of the Rings stories.

The last two questions were control questions, to make sure there was no sampling bias in the subjects enjoyment or familiarity with the fictional setting of the Lord of the Rings.

The standard in our course is to require the students to turn in a rough draft of the worksheet before the tutorial time. The rough draft is only graded for completion, not accuracy. That way, students must engage with the problems and can identify parts of the assignment that are confusing or difficult, and then we can spend tutorial time focused on those parts. The final draft of the worksheet is due at the end of the week, after all the tutorial sessions. Attendance at the tutorial sessions is optional. Students can also discuss the worksheets in a Slack channel dedicated to their tutorial session. Since sharing of ideas and collaboration is common and even encouraged on the worksheets, we did not use the grades on the worksheet as a primary outcome. We told the students that we were testing two possible articles and worksheets on life history theory and requested that they not share the article or worksheet with peers in other tutorial sections. We also told them that if one article proved statistically significantly better than the other based on their exam scores, we would curve up the scores of the students who had gotten the worse article so that their grade would not suffer from the exercise. In the end, 123 students turned in the worksheet for the fiction condition and 114 students turned in the worksheet for the fact condition.

Of the eight questions, worth a total of 8 points, on life history theory on the exam (see Additional file 4: Text S4), the 131 students who were randomly assigned to the fiction condition scored an average of 4.95 points (Std.Dev. = 1.83), and the 133 students who were randomized to the fact condition scored an average of 4.94 points (Std.Dev. = 1.75), which was not statistically significantly different from the fiction condition (two-tailed T-test, *p* = 0.95). However, among the students who read the fiction article, the degree to which they liked The Lord of the Rings was associated with performing better on the life history questions on the exam (linear regression coefficient = 0.38, *p* = 0.004). Students who liked The Lord of the Rings more but read the factual article showed some evidence of doing better on the exam though it was not quite statistically significant (linear regression coefficient = 0.23, *p* = 0.06), suggesting that they might be more engaged in general, learned more from our lectures, or were stronger students for some other reason.

**Fig. 6:**
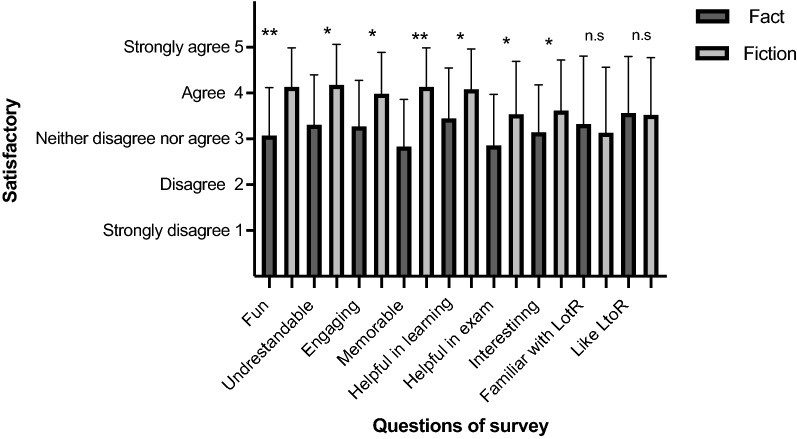
Survey of reader experiences. There were statistically significant differences in the 5–point Likert scale for all the readers experience questions (*p < 10^−3^, **p < 10^−5^ for T-tests). The final 2 questions were control questions which show that there were no significant differences (non-parametric Wilcoxon rank sum test, *p* > 0.3) in the students’ familiarity and liking of the Lord of the Rings stories

**Fig. 7:**
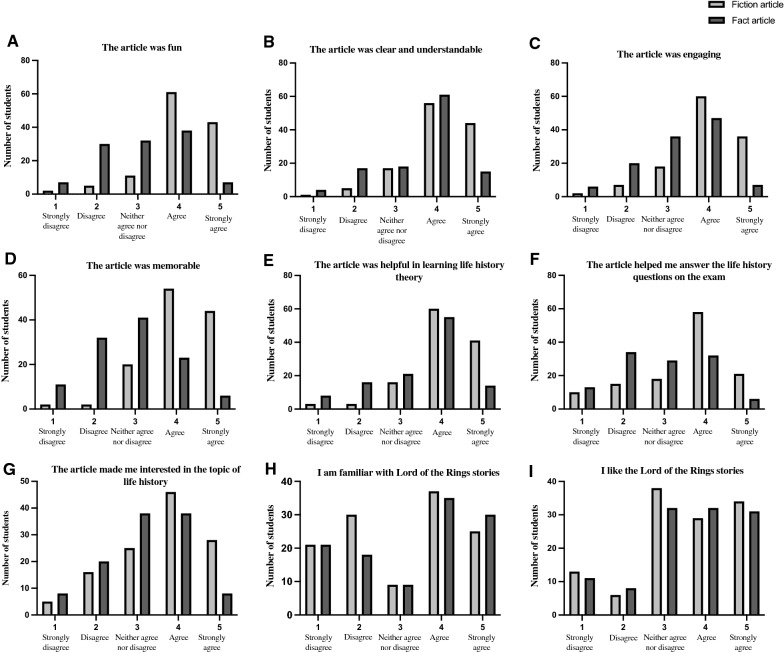
The distribution of responses for each question on the reader experience survey. Responses for the fiction article are in light gray and for the factual article in dark gray

When we surveyed their experience of reading the articles, the students in the fiction condition found the reading more fun (Figs. 6 and 7A, T-test *p* < 10^–5^), more clear and understandable (Figs. 6 and 7B, T-test *p* < 10^–3^), more engaging (Figs. 6 and 7C, T-test *p* < 10^–3^), more memorable (Figs. 6 and 7D, T-test *p* < 10^–5^), more helpful in learning life history theory (Figs. 6 and 7E, T-test *p* < 10^–3^), they perceived it as more helpful in answering the life history questions on the exam (Figs. 6 and 7F, T-test *p* < 10^–3^) even though they did not actually perform better on those exam questions, and they reported that the fiction reading made them more interested in life history theory compared to students in the fact condition (Figs. 6 and 7G, T-test *p* < 10^–3^). These effects were all highly statistically significant under non-parametric Wilcoxon rank sum tests (Additional file 5: Table S1). Furthermore, there were statistically significant interactions between the degree to which students liked The Lord of the Rings and how fun, engaging and memorable they found the fiction article, as well as how much it made them interested in life history theory and how much they thought it helped them prepare for and perform on the exam (linear regressions *p* < 10^–4^, Additional file 6: Table S2), though there was no association between liking The Lord of the Rings and how clear and understandable they found the article (*p* = 0.86). There were no significant differences in the degree of familiarity with Lord of the Rings in the two groups or the extent to which they liked The Lord of the Rings (Figs. 6 and 7H, I, Wilcoxon rank sum test *p* > 0.35), though there was a strong correlation between familiarity with and liking of The Lord of the Rings (*r* = 0.84, *p* < 10^–16^). Mean values and p-values for each question are provided in Additional file 7: Table S3.

## Discussion

Reading the fictional article rather than the factual article did not detract from the students education, as the two two groups scored the same on the exam. In fact, those students who liked The Lord of the Rings and read the article on the life history theory of Middle Earth actually performed better on the exam, and for all the students there were large and highly statistically significant positive effects on their enjoyment, engagement and interest in life history theory from reading the article on the Lord of the Rings. Similar results have been found in other uses of fiction for teaching science (Vrasidas et al. 2015). Using fiction may affect long term retention of the materials, and hopefully, retention of students in science, but we have not yet measured that.

As an in-class exercise, we often ask students to choose a favorite fictional species (from any culture or background) and then note down what life history characteristics are known about that species, as well as hypothesize likely characteristics that were never described but can be inferred from life history theory. They tend to respond enthusiastically to this exercise and we believe it is valuable to help them generalize the concepts of life history theory to a novel concept while also engaging their imaginations. The fact that fans of Lord of the Rings benefitted the most from our Lord of the Rings examples, suggests that more students could benefit from generalizing scientific ideas to their favorite stories from their own cultures. In this way, our teaching could be both more inclusive and more effective. In the future, we plan to use this article to teach life history theory, but to focus the worksheet questions on stories chosen by each student to tap into their own particular passions.

We are not arguing for the replacement of all factual articles with fictional narratives in science education. Students must learn how to read, critique and write scientific articles. However, as science is an endeavor to discover what is not already known or understood, we believe the exercise of imagination and the dreaming of what may be are also fundamental skills of a good scientist. Engagement with fiction is one way to help nurture those skills.

## Conclusions

Tolkien believed that there is a form of truth in myths (Carpenter 1977). One of the things that make fictional worlds compelling is the degree of internal consistency in those worlds. Life history theory helps to provide such internal consistency for the ecology of fictional worlds, and at the same time, fiction gives us a playground in which to explore patterns in the real world. The ability to generalize a scientific concept to a new context represents one of the deepest levels of understanding (Crowe et al. 2008). Fiction provides both novel and compelling contexts to explore scientific ideas. We hope that this approach to teaching science (Bixler 2007) will be useful for other teachers and also inspire non-traditional students, like those interested in fictional world-building, beyond our traditional institutions of education.

## Supplementary Information

**Additional file 1: Text S1.** The body of this article, without the experimental evaluation section, provided for teachers to use to teach life history theory.

**Additional file 2: Text S2.** A six question worksheet for the fiction article.

**Additional file 3: Text S3.** An equivalent six question worksheet for the factual article.

**Additional file 4: Text S4.** Eight multiple choice questions used for our exam.

**Additional file 5: Table S1.** Results of non-parametric Wilcoxon matched-pairs signed rank tests of survey questions show the same statistically significant results as the T-tests.

**Additional file 6: Table S2.** Linear regression results using the degree to which students like Lord of the Rings (Q9) to predict their answers to the other questions on the survey.

**Additional file 7: Table S3.** Mean values of Likert scale responses for the reader survey.

**Additional file 8: Table S4.** Anonymized individual exam scores for each student in each condition.

## Data Availability

The preprint version of this article (minus the experimental section) that was used in fiction condition is available in the additional files, as well as the worksheets that were used in both conditions, and the exam questions that we used. The anonymized test results and survey responses are available in Additional file 8: Table S4.

## Footnotes

1 Though Arwen herself carries some human genes through her father’s side.
