# Supplementary material for: The life history theory of the Lord of the Rings: a randomized controlled trial of using fact versus fiction to teach life history theory
Source: Evolution (N Y). 2022 Feb 16;15(1):2. doi: 10.1186/s12052-022-00160-8 (PMC8850221; doi:10.1186/s12052-022-00160-8)
Supplement: Supplementary file 1 — Additional file 1: Text S1. The body of this article, without the experimental evaluation section, provided for teachers to use to teach life history theory. [file 12052_2022_160_MOESM1_ESM.docx]

**The Life History Theory of the Lord of the Rings**

Carlo C. Maley^†^ and Sareh Seyedi

Arizona Cancer Evolution Center, Biodesign Institute and School of Life Sciences, Arizona State University

^†^ corresponding author

# Introduction: Science Fantasy for Science

One of the key skills we hope to build in our students is the ability to generalize principles to a novel context. This is central to the process of science, and one of the key drivers of innovation and interdisciplinary research. Such generalization demonstrates a deep understanding of the principle, rather than a simple recall of the examples the student has previously seen. In order to challenge students to generalize concepts to new contexts, teachers can borrow examples from nature that have not been previously covered in class. However, it can also be a powerful motivator and inspiration to apply ideas to examples from fantasy and science fiction that have already shown success in firing students’ imaginations [(1)](https://paperpile.com/c/iKvXOj/Uez2d), and to utilize the narratives of those stories to enhance education [(2)](https://paperpile.com/c/iKvXOj/zxVuK). The Lord of the Rings has been used previously as an analogy to teach the human immune response [(3)](https://paperpile.com/c/iKvXOj/hYead). Here, we apply this technique to teach life history theory using the creatures of J.R.R. Tolkien’s Middle-Earth.

But first we must acknowledge that Tolkien was not perfect. He was a wonderful philologist and storyteller, but he was not a biologist [(4#153)](https://paperpile.com/c/iKvXOj/KYCPH/?suffix=%23153). One of his express goals in his work was to provide Britain with its own mythology [(5)](https://paperpile.com/c/iKvXOj/0C8xp). Thus, we must take care to separate his keen observations from his myth making. This is most clear in his observations about elves. He claims that they are immortal [(6 Appendix A)](https://paperpile.com/c/iKvXOj/kzuAI/?suffix=Appendix%20A), which is biologically implausible. We may safely dismiss this as mythologizing and the exaggeration of an extremely long lifespan.

Though Tolkien left behind many pages of notes on Middle-Earth, there is only so much that a single observer can catalog. We owe a debt to his son Christopher for collating, editing and publishing those notes that provide our only knowledge of Middle-Earth [(7–10)](https://paperpile.com/c/iKvXOj/4dlBo+dzsnG+T0mJF+wzulv). For the rest, for the gaps in Tolkien’s observations, we may apply life history theory to infer and predict many undocumented aspects of the hominids (elves, orcs, dwarves and hobbits) of Middle-Earth.

The life history traits of a species are those characteristics that determine the organism’s likelihood of survival to a given age, and the number of offspring it typically produces at each age. These include traits like lifespan, growth rate, age of sexual maturity, frequency of reproduction, number of infants per birth (e.g., twins), susceptibility to infection, body size, and traits that affect the ability to compete for mates and resources. These traits are shaped by the selective pressures of the ecology of a species, which often leads to correlations between different life history traits. For example, large organisms often live longer, delay reproduction and have fewer offspring than small organisms.

# Hominids of Middle-Earth

The hominids of Middle-Earth show extreme variation for many life history traits. What is known about the hominids of Middle-Earth and what can we infer from that?

## Elves


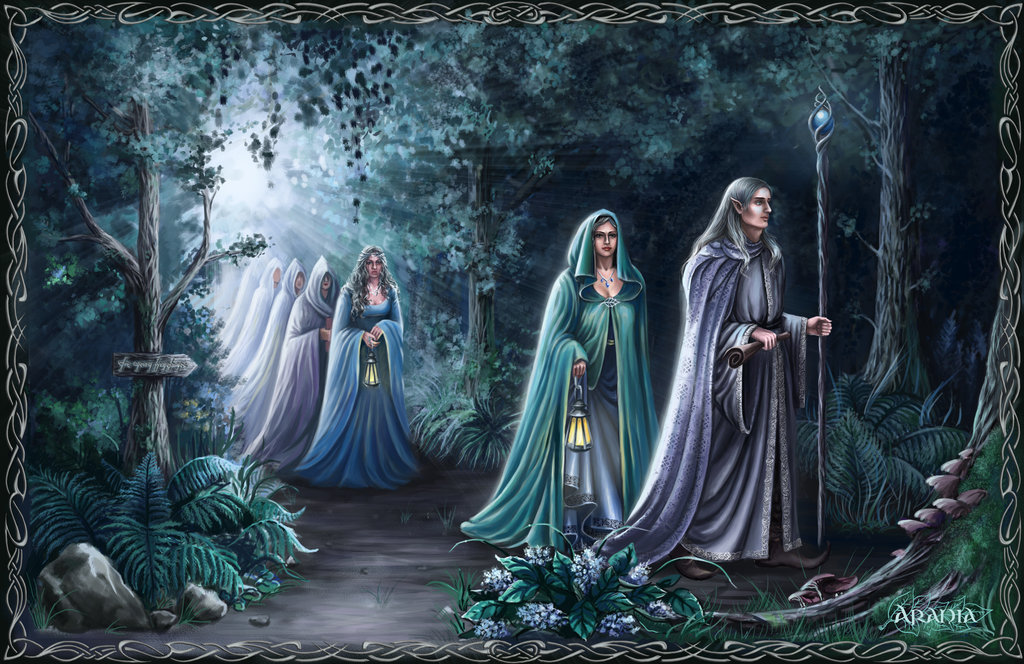


*Figure 1. An artist’s rendition of elves. Image by Araniart / CC BY (https://creativecommons.org/licenses/by/3.0)*

The elves of Middle-Earth (Figure 1), are tall, almost ethereal creatures of extremely long lifespans . Though they can walk, talk and dance by one year of age, they take 50-100 years to fully mature [(11)](https://paperpile.com/c/iKvXOj/6m08n). Elves have few children, and long interbirth intervals [(11)](https://paperpile.com/c/iKvXOj/6m08n). Tolkien states that they are immortal [(6 Appendix A,11)](https://paperpile.com/c/iKvXOj/kzuAI+6m08n/?suffix=Appendix%20A,), in that their faculties do not decline with age (i.e., senesce) [(4#153)](https://paperpile.com/c/iKvXOj/KYCPH/?suffix=%23153), but we may take this as an exaggeration of an incredibly long lifespan. Though they can be killed by violence, they have better wound healing abilities, resistance to disease and stamina compared to humans [(12 Note 5)](https://paperpile.com/c/iKvXOj/akXrR/?suffix=Note%205). The combination of long life, disease resistance and effective wound healing should not be surprising. If there has been natural selection on elves to evolve extremely long lifespans, the same ecological conditions would select for the prevention of premature death from wounds or disease.

What are those ecological conditions that select for a long lifespan? If there is very little risk of death from predators, competitors or diseases, called “external sources of mortality”, then populations will grow until they become limited by resources. At that point, selection shifts from external sources of mortality to competition for limited resources. Competition for limited resources usually selects for the evolution of what are called slow life history strategies. These include a constellation of traits including long lifespans, large body size, high levels of investment in maintaining those bodies, few offspring, long periods of development before reaching sexual maturity, and high levels of parental investment in those offspring. Elves’ lifespan, height, slow growth to maturity, and superior wound healing and disease resistance are all consistent with elves exhibiting the slowest life history strategy known in mammals. It may only be rivaled by some trees on Earth, such as bristlecone pine trees, that can live thousands of years [(13)](https://paperpile.com/c/iKvXOj/7l0LY).

What does all of this imply about elves that was not documented by Tolkien? Long interbirth intervals and few children implies that there must be very high levels of parental investment in their elflings. Matings and births must be very important events in elven culture. Long lifespan is also strongly selected in species for which older individuals are better able to compete for mates and status, and otherwise successfully reproduce. This suggests that older elves likely have higher status and more successful matings than younger elves. We might question Tolkien’s claim that elves tend to monogamously marry and produce offspring shortly after attaining sexual maturity, losing interest in sex thereafter [(11)](https://paperpile.com/c/iKvXOj/6m08n).

Elves’ extremely long lifespans suggest that they were able to effectively avoid death from conflict with the other hominids of Middle-Earth, as well as other elves, during most of their evolution. They do appear to live in extremely well guarded enclaves in Middle-Earth [(14)](https://paperpile.com/c/iKvXOj/qpMVL). This also implies that they must expend a great deal of their resources and time on limiting risks to themselves. This may help to explain their insular nature, their high levels of competence at various survival skills, and their preference for use of bow and arrow over more risky forms of close combat. Their long lifespans and investment in somatic maintenance suggests that, like elephants, they probably also have effective mechanisms for dealing with DNA damage and preventing cancer [(15)](https://paperpile.com/c/iKvXOj/aP1qX).

Elves’ very long lifespans and slow reproduction calls into question why they would ever risk leaving their safe enclaves. One possibility is that there has been kin selection for sending out scouts to identify potential threats. The risk of scouting to the individual scout might be outweighed by the survival benefit to its relatives by identifying and defusing external threats, thereby preserving and spreading the alleles of genes in the relatives that made the scouts willing to take that risk. Another possibility is that leaving the enclave is a potential high risk / high payoff strategy for acquiring resources or prestige which would likely translate into increased reproductive opportunities. Given their long evolutionary history where competition for resources was probably the primary selective pressure, it would not be surprising if there has been sexual selection on elves to choose mates that have proven effective at acquiring resources. In contrast, there is evidence that there is little competition between males or between females (intra-sexual competition). Such intra-sexual competition often leads to the evolution of sexual dimorphism (physical differences between the sexes of a species), as seen in the large antlers male moose, but not female moose, grow every year. However, elves have even less sexual dimorphism than humans[(11)](https://paperpile.com/c/iKvXOj/6m08n), who themselves have less sexual dimorphism than most primates[(16,17)](https://paperpile.com/c/iKvXOj/pRpME+jWXs2). The lack of sexual dimorphism suggests that there is little reproductive skew in elves. That is, even high status elves probably do not monopolize multiple mates. This is supported by Tolkien’s observation that they are highly monogamous [(11)](https://paperpile.com/c/iKvXOj/6m08n).

## Orcs (a.k.a., Goblins, Uruk)

##
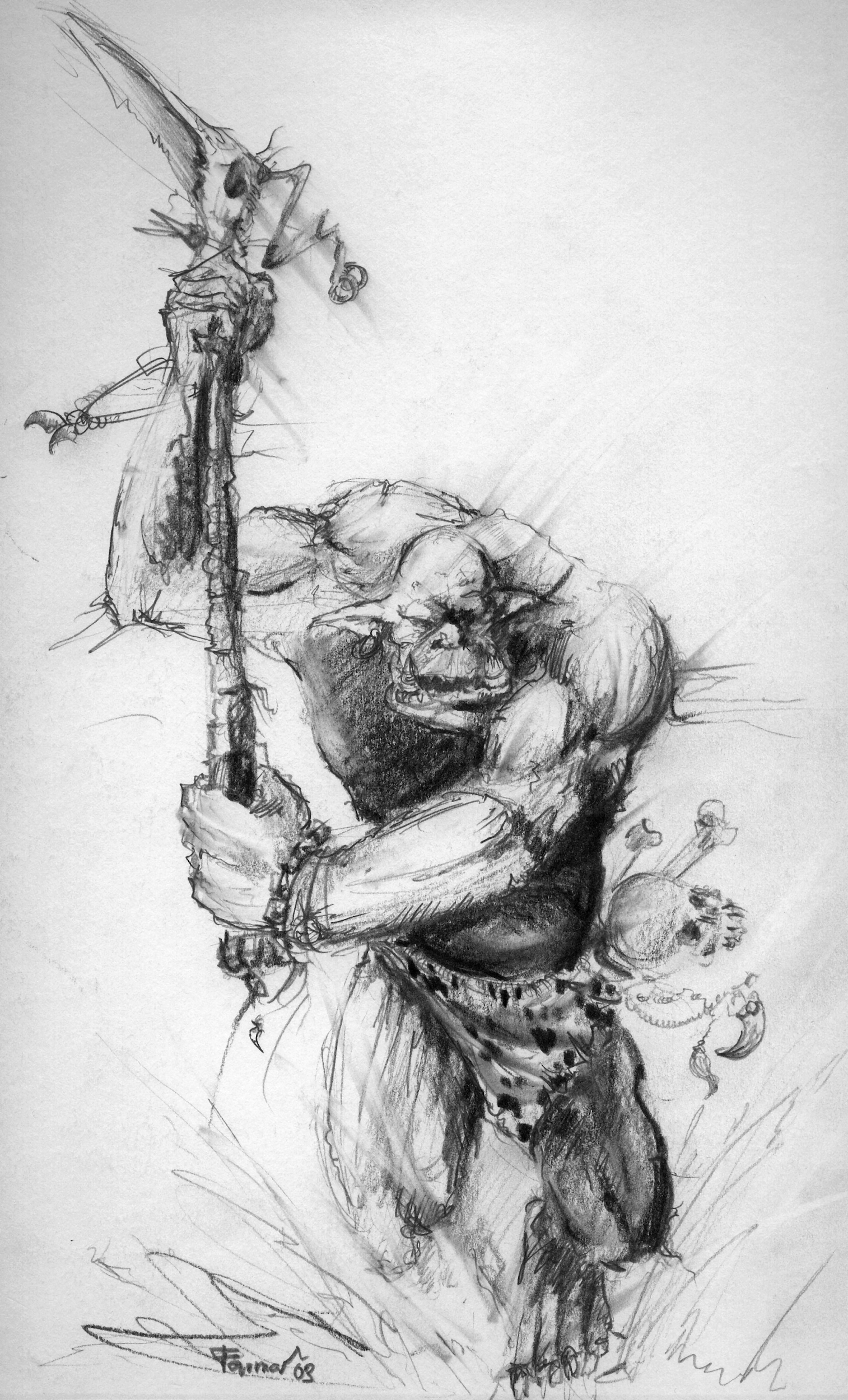


*Figure 2. An artist’s image of an orc. Given the small size of most orcs, the skull on its belt probably belonged to another orc or a hobbit. Alternatively, this might be a reproduction of an Uruk-hai, one of the larger orcs. Image by farmerownia (www.farmerownia.pl) (www.faun-forge.com) / CC BY-SA (https://creativecommons.org/licenses/by-sa/3.0)*

The orcs of Middle-Earth (Figure 2) are small [(6 Appendix A,18)](https://paperpile.com/c/iKvXOj/kzuAI+NBSXS/?suffix=Appendix%20A,), so small in fact that 3-foot (1 meter) tall hobbits in disguise can pass for orcs [(6 The Land of Shadow)](https://paperpile.com/c/iKvXOj/kzuAI/?suffix=The%20Land%20of%20Shadow). They are generally squat and broad [(4#210)](https://paperpile.com/c/iKvXOj/KYCPH/?suffix=%23210), mostly live underground and are sensitive to sunlight [(18)](https://paperpile.com/c/iKvXOj/NBSXS). Orcs have short lifespans and rapidly multiply in their enclaves [(19,20)](https://paperpile.com/c/iKvXOj/jBFNT+7rZtU). In short, they have evolved a fast life history strategy. And it is no wonder, for in contrast to elves, there is clearly a great deal of external mortality for orcs. They are not only hunted down and killed by the other hominids of Middle-Earth, but there is intense violence between orcs and conflict over status that is often lethal [(6,18)](https://paperpile.com/c/iKvXOj/NBSXS+kzuAI). Any species that experiences such high levels of mortality is under selection to reproduce quickly before they are killed. This means that they must reach sexual maturity quickly, which generally selects for a smaller body size, as does living underground.

The violent clashes between orcs over hierarchy suggest that orcs probably have extreme reproductive skew, with high status males monopolizing mating opportunities. It is unclear if there is also mate competition among female orcs. Unfortunately, Tolkien did not observe any female orcs, and so we know little about them. If they are like other fast life history mammals, high mortality rates would have led to the evolution of more than two mammary glands in female orcs, large numbers of offspring, probably with multiple infants per birth. In the extreme, selection for fast life histories can lead to semelparity - organisms that reproduce only once, but massively, in their life and then die. It is unclear if orcs are semelparous, but it seems more likely that they would reproduce like other fast life history mammals, such as mice, that repeatedly have large litters. Selection for fast life history strategies also selects for low levels of parental investment. This may have led to the evolution of low levels of oxytocin and affiliative bonding between orcs, which would be consistent with descriptions of their interactions [(6,18)](https://paperpile.com/c/iKvXOj/NBSXS+kzuAI).

Boom and bust cycles, and other causes of large population fluctuations, also select for fast life histories. If there is a sudden expansion of resources, or the population is knocked down well below the carrying capacity of the environment, then individuals who can reproduce the fastest will fill that vacuum and dominate future generations. We know that a number of wars caused large population fluctuations in orcs. In fact, the War of Wrath that ended the first age almost led to their extinction [(7)](https://paperpile.com/c/iKvXOj/4dlBo). Such significant population bottlenecks tend to lead to high frequencies of genetic diseases.

If an orc survived to old age, it probably would die of cancer. In species that suffer a high degree of external mortality and have little parental investment, there is no selection for maintaining the body late in life, including cancer suppression. They also likely suffered from high levels of infectious disease, for the same reason. Fast life history organisms that devote the majority of their resources to rapid growth and reproduction will out-compete those that devote their resources to a strong immune system and cancer suppression, only to be killed through violence. The one exception might be wound healing. Given the levels of violence in the experience of orcs, they were probably selected for effective wound healing.

One common feature of fast life history organisms is that they tend to disperse widely, like weeds that persist by quickly colonizing new disturbances in the environment. Small body size and low investment in their bodies might render orcs at a disadvantage when directly competing with other hominids of Middle-Earth, which is supported by the fact that they lost most of the documented wars with the other hominids over the ages. So instead of directly competing, there would have been selection on orcs to tend to migrate in order to find environments where they could thrive without direct contact with humans, elves or dwarves.

Finally, we should note that orcs are clearly not the unthinking brutes that they were often portrayed as. The fear and long standing deadly conflict between orcs and the other hominids of Middle-Earth probably biased reports of their nature. The size and organization of their armies, including forged weapons and armor (regardless of their poor quality), as well as complex machinery of war (e.g., catapults) and mining (including engines and explosives) [(21)](https://paperpile.com/c/iKvXOj/ZjFs2), speaks to a highly developed civilization and industry.

## Dwarves


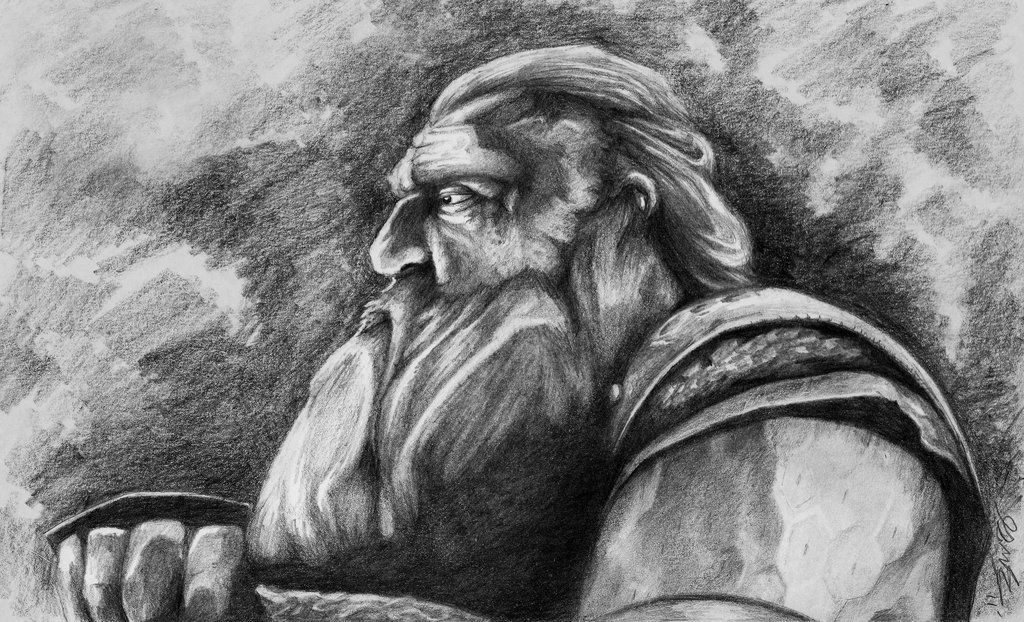


*Figure 3. An artist’s drawing of a dwarf, probably male. Image by Perrie Nicholas Smith / CC BY-SA (https://creativecommons.org/licenses/by-sa/4.0)*

Dwarves are the mortal enemies of orcs, and very different from them biologically (Figure 3). Dwarves have very long lifespans, averaging around 250 years, with very little senescence until the last decade of their life [(22)](https://paperpile.com/c/iKvXOj/VwS94). They take 30 years to grow to adulthood and do not usually reproduce before 90 years of age [(6 Appendix A)](https://paperpile.com/c/iKvXOj/kzuAI/?suffix=Appendix%20A). In other words, they have a much slower life history strategy than orcs or even humans. They are short and generally live underground, though they eat the same kind of foods as humans and hobbits [(21,23)](https://paperpile.com/c/iKvXOj/wVP1U+ZjFs2). They are resistant to most diseases, except obesity [(22)](https://paperpile.com/c/iKvXOj/VwS94), and are even resistant to damage from fire [(7)](https://paperpile.com/c/iKvXOj/4dlBo). Dwarves can withstand suffering and toil more than the other hominids of Middle-Earth [(7)](https://paperpile.com/c/iKvXOj/4dlBo). This all adds up to a clear picture of a slow life history strategy for dwarves. This is pretty typical for creatures that live underground [(24)](https://paperpile.com/c/iKvXOj/49ZUA), when living underground provides a good defense against predators and other sources of violent death. However, life history strategies can be flexible and respond to signals from the environment. Given the well documented population fluctuations of dwarves, due to wars [(6 Appendix A,7)](https://paperpile.com/c/iKvXOj/kzuAI+4dlBo/?suffix=Appendix%20A,) and dragon attacks [(21)](https://paperpile.com/c/iKvXOj/ZjFs2), it is quite likely that dwarves can shift into a faster life history strategy to exploit the newly available space and resources after such a population bottleneck.

Dwarves do not generally farm for themselves and there are no mentions of them being hunter-gatherers [(7)](https://paperpile.com/c/iKvXOj/4dlBo). Because Dwarves live underground but depend on food grown above ground, they must form symbiotic relationships with other hominids to supply their food. Indeed there are multiple reports of such relationships with humans [(6 Appendix A)](https://paperpile.com/c/iKvXOj/kzuAI/?suffix=Appendix%20A), elves [(7)](https://paperpile.com/c/iKvXOj/4dlBo) and even hobbits [(23)](https://paperpile.com/c/iKvXOj/wVP1U). Those relationships go beyond trade for food. Dwarves are expert miners and smiths who provide high quality arms and armor to both elves and humans who then fight off their mutual enemies [(7)](https://paperpile.com/c/iKvXOj/4dlBo). Although there is little mention of it, we might expect dwarves to protect their suppliers of food and allies in the dwarven strongholds during times of strife.

Dwarves are unusual in that there are twice as many males as females [(6 Appendix A)](https://paperpile.com/c/iKvXOj/kzuAI/?suffix=Appendix%20A). This sex bias is thought to slow their population growth rate [(6 Appendix A)](https://paperpile.com/c/iKvXOj/kzuAI/?suffix=Appendix%20A). Normally, such a skewed sex bias would lead to intense competition among the males for mating opportunities, and that intra-sexual competition often leads to sexual dimorphism. However, dwarves are notorious for their lack of sexual dimorphism. In fact, female dwarves look so much like male dwarves, including sporting beards, that they are often mistaken for males [(6 Appendix A)](https://paperpile.com/c/iKvXOj/kzuAI/?suffix=Appendix%20A). Given the sex bias, one might expect dwarves to be polyandrous, with females taking multiple male mates, but this does not appear to be the case. Dwarves are generally monogamous, taking only one husband or wife in their lives, and are jealous of both wealth and their mates [(6 Appendix A)](https://paperpile.com/c/iKvXOj/kzuAI/?suffix=Appendix%20A). Instead of male-male competition or polyandry, many male dwarves opt out of competing for mates and prefer to focus on their craft. This is reminiscent of many species in which relatives forgo their own reproduction to help with the care and survival of their close kin, sometimes called “helpers in the nest”[(25)](https://paperpile.com/c/iKvXOj/9Tbz9). Since their kin also carry the alleles for such non-reproductive helping behavior, those alleles can spread in the population. This is a phenomenon called kin selection.

The dwarves’ obsession with treasure, and particularly beards, suggests that sexual selection among dwarves focuses on those traits. In fact, dwarven beards come in a variety of colors, from yellow, to blue, to white, like the colorful plumage of birds that appears to have evolved through sexual selection [(26)](https://paperpile.com/c/iKvXOj/8plBj). Luxuriant beards may also help to advertise a low disease burden and thus a desirable mate, an idea called the handicap principle [(27,28)](https://paperpile.com/c/iKvXOj/OXxs7+nwKTw). Similarly, sexual selection for mates with abundant resources would explain dwarves’ lust for gold and their motivation to leave their safe underground dwellings to seek treasure[(21)](https://paperpile.com/c/iKvXOj/ZjFs2). The focus on treasure is likely also explained by dwarves’ dependency on trade with other hominids to supply them with food and other necessities that they cannot procure themselves. It is no wonder that they have invested so much effort and skill in mining for precious metals and gems, as well as crafting highly desired tools and artefacts from them.

## Hobbits


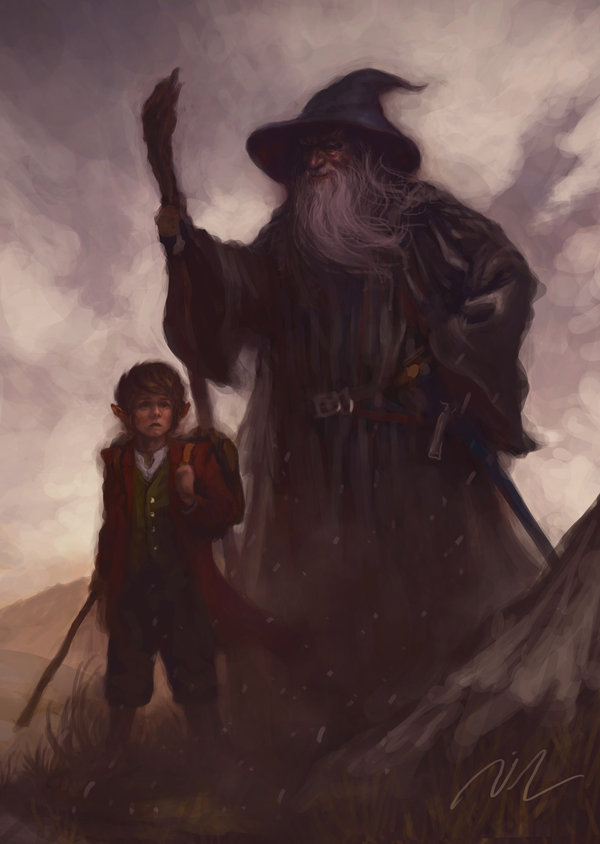


*Figure 4. An artist’s rendition of a hobbit. Shown here with a human sized wizard, for scale. Image by Joel Lee (maxbat) / CC BY-SA (https://creativecommons.org/licenses/by-sa/4.0)*

Hobbits, called halflings by humans, are short, usually between 2 and 4 feet tall (around 1 meter) [(14 Prologue)](https://paperpile.com/c/iKvXOj/qpMVL/?suffix=Prologue) (Figure 4). They traditionally live underground in cozy dwellings of large, multi-generational families, though some now live above ground in houses that maintain some of the architecture of a hobbit hole. They have large families and live a little longer than humans, averaging approximately 100 years [(14)](https://paperpile.com/c/iKvXOj/qpMVL). They are exceptionally fond of food and drink. They typically consume six meals a day (seven if you count “second breakfast”) [(14 Prologue)](https://paperpile.com/c/iKvXOj/qpMVL/?suffix=Prologue). The fact that they consume so much food may well limit the carrying capacity of their environment and explain in part their relatively limited population size, compared to humans.

They are shy of humans and other “big folk,” and are excellent at hiding from them [(14 Prologue)](https://paperpile.com/c/iKvXOj/qpMVL/?suffix=Prologue). They are neither prone to war nor violent strife among themselves [(14 Prologue)](https://paperpile.com/c/iKvXOj/qpMVL/?suffix=Prologue). They seem to be protected from the violence of the other hominids by the service of the human rangers and at least one wizard [(14)](https://paperpile.com/c/iKvXOj/qpMVL), suggesting that they are good at coopting the investment of other hominids of Middle-Earth. They also have strong social norms that encourage proper behavior and discourage risk taking as well as travel. Most of this speaks of a slow life history strategy, though not as slow as dwarves or elves. This is consistent with few sources of external mortality for hobbits. For any slow life history species, the cost of risks is higher than for fast life history species, as the slow life history organisms must generally survive for a long time to attain resources and mates, as well as rear their young. This may explain hobbits’ aversion to risk.

Unlike most slow life history species, hobbits do have many children and only grow to a small size. However, their multigenerational families suggest significant parental investment in their young. Their small size probably evolved in part as an adaptation to underground living rather than adult mortality, though they clearly spend a fair amount of time farming above ground. Their slow life history traits suggest that hobbits probably have excellent defenses against both infectious disease and cancer.

Are humans, elves, ocrcs, dwarves, and hobbits different species, or just different variations within a single species? There is evidence for both alternatives (Box 1).

# The lessons of life history theory and evolutionary ecology

The ecology of a species shapes its life history traits, through natural selection. Many of those traits are correlated and are often described as forming a continuum from fast to slow life history strategies (Figure 5). It is not the case that one life history strategy is better than another, per se. Which life history strategy works best depends on the environment and the other species in that environment. If members of a species are rapidly and often killed off, perhaps by predators, or maybe environmental disturbances like fires and droughts, then those individuals that can reach sexual maturity quickly and reproduce rapidly, producing large litters, will tend to leave behind more offspring than the individuals that grow more slowly, investing in a robust body only to be killed before they can reproduce.


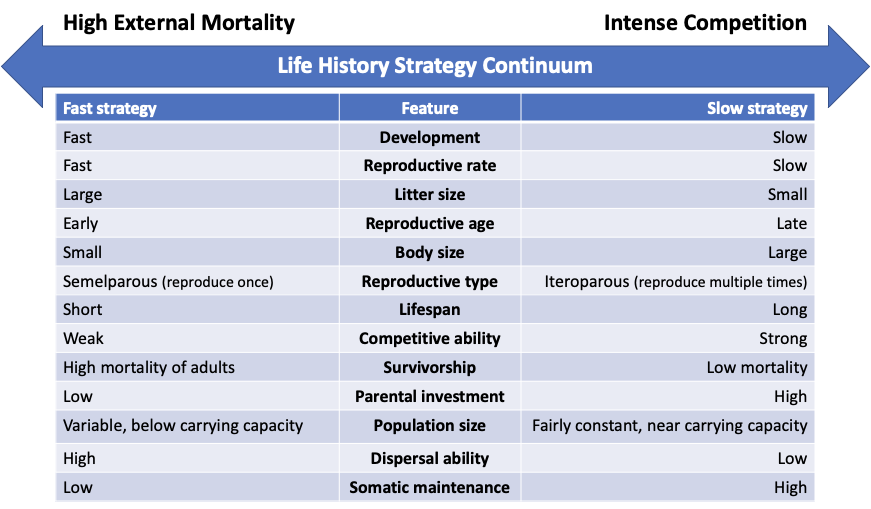


*Figure 5. Life history strategy continuum. Ecologists often put life history strategies on a continuum from fast to slow. Selection due to high levels of external mortality (like predation) select for fast life history strategies while low levels of external mortality but high levels of competition within a species tend to select for slow life history strategies.*

In contrast, if there is very little external mortality, then a population will tend to grow until it reaches some limitation of resources. At that point, the organisms that can most effectively compete for those limiting resources will tend to survive and reproduce better than their ineffective competitors. These ecologies select for organisms that can build robust bodies and can live a long time (Figure 5), fending off diseases. They also select for organisms that invest a lot of resources in their young, protecting them and raising them until they can effectively compete with other members of their species. Because resources are limited, this generally means that organisms with such slow life history strategies grow slowly and have few offspring. There are a variety of ways that a species might evolve to avoid predation including growing large [(29)](https://paperpile.com/c/iKvXOj/59ZM5), living underground [(24)](https://paperpile.com/c/iKvXOj/49ZUA), or evolving flight [(30)](https://paperpile.com/c/iKvXOj/8Ew5S). This helps to explain why bats have evolved to live so much longer (e.g. 40 years for Brandt’s bat [(31)](https://paperpile.com/c/iKvXOj/wOCJH)) than other mammals of the same size. Even within the same environment, there can be different niches where different life history strategies can thrive. For example, fast life history weeds and rodents often coexist in forests with slow life history trees, bats, and birds. In addition, many organisms can shift their life history strategies, and even switch between sexual and asexual reproduction, in response to signals from their environment [(32,33)](https://paperpile.com/c/iKvXOj/riadi+hHC16).

Sexual selection can also have strong effects on the evolution of species. This includes one sex choosing which individuals of the other sex to mate with (inter-sexual competition) as well as members of one sex competing with each other for mating opportunities (intra-sexual competition). If there is a lot of reproductive skew, if a minority of individuals in a population get all the mating opportunities, then competition to be in that reproductive minority can be intense, and selection for traits that provide for success in such competition can swamp other selective pressures (like cancer suppression [(34)](https://paperpile.com/c/iKvXOj/RxWLu)).

## Box 1: Are the hominids of Middle-Earth different species?

With such different life history strategies, we might ask if the different hominids of Middle-Earth are different species. Biologists generally define a species as a reproductively isolated population [(35)](https://paperpile.com/c/iKvXOj/QjkWw). That is, two populations are different species if they cannot interbreed, producing fertile offspring. Clearly, elves and humans may successfully interbreed, producing fertile half-elves in at least three documented unions (Idril and Tuor; Luthien and Beren; Arwen^^[[1]](#footnote-0)^^ and Aragorn)[(6 Appendix A)](https://paperpile.com/c/iKvXOj/kzuAI/?suffix=Appendix%20A). Elrond the half-elven was the grandson of both Idril and Tuor as well as Luthien and Beren. Elrond himself is fertile, having produced two sons, Elladan and Elrohir, as well as a daughter, Arwen, who in turn was able to successfully breed with a human, Aragorn, son of Arathorn. So humans and elves appear to be different morphs of the same species with extremely divergent life history strategies. Tolkien supports this view in a letter stating that “Elves and Men are just different aspects of the Humane.” [(4#181)](https://paperpile.com/c/iKvXOj/KYCPH/?suffix=%23181)

Orcs can also interbreed with humans [(19)](https://paperpile.com/c/iKvXOj/jBFNT). There are suggestions of admixture of human genes into the fighting Uruk-hai that Saruman bred, making them larger than normal orcs and allowing them to operate comfortably in daylight [(19)](https://paperpile.com/c/iKvXOj/jBFNT). Saruman’s armies also included half-orcs [(18)](https://paperpile.com/c/iKvXOj/NBSXS). In addition, there are references to half-orcs that Saruman bred and used as spies [(14)](https://paperpile.com/c/iKvXOj/qpMVL) and guards [(6 Appendix A)](https://paperpile.com/c/iKvXOj/kzuAI/?suffix=Appendix%20A). Legend has it that orcs were either derived from elves [(7)](https://paperpile.com/c/iKvXOj/4dlBo) or from humans [(19)](https://paperpile.com/c/iKvXOj/jBFNT), and so it is likely that orcs, humans and elves are all part of a single species, spanning extreme ranges of life history strategies.

Despite hobbits being more closely related to humans than humans are to elves [(14 Prologue)](https://paperpile.com/c/iKvXOj/qpMVL/?suffix=Prologue), there is no mention of successful matings between hobbits and humans, or between hobbits and any of the other hominids of Middle-Earth. Similarly, there is no mention of successful matings between dwarves and other hominids, suggesting true species barriers between them and the other hominids of Middle-Earth. In fact, hobbits may be in the process of dividing into three species as the three hobbit breeds (Harfoots, Stoors and Fallohides) have evolved different physical characteristics, which suggests limited interbreeding between the hobbit breeds. This reproductive isolation was probably reinforced by their preferences for different habitats: Harfoots in the highlands and hillsides, Stoors in the flatlands and riversides, and Fallohides in forests [(14 Prologue)](https://paperpile.com/c/iKvXOj/qpMVL/?suffix=Prologue).

Colin's predicted relationships between Tolkien’s hominids using a character based phylogenetic reconstruction method [(36)](https://paperpile.com/c/iKvXOj/V1GI). His phylogeny groups orcs and elves together (due to their pointed ears and loss of facial hair), suggesting that they are more closely related to each other than they are to humans. Though his analysis suggests that Dwarves and hobbits are more closely related to humans than they are to elves and orcs, the evidence of reproductive barriers does not support this.

**Conclusions**

Tolkien believed that there is a form of truth in myths [(5)](https://paperpile.com/c/iKvXOj/0C8xp). One of the things that make fictional worlds compelling is the degree of internal consistency in those worlds. Life history theory helps to provide such internal consistency for the ecology of fictional worlds, and at the same time, fiction gives us a playground in which to explore patterns in the real world. The ability to generalize a scientific concept to a new context represents one of the deepest levels of understanding [(37)](https://paperpile.com/c/iKvXOj/lNKE3). Fiction provides both novel and compelling contexts to explore scientific ideas. We hope that this approach to teaching science [(1)](https://paperpile.com/c/iKvXOj/Uez2d) will be useful for other teachers and also inspire non-traditional students, like those interested in fictional world-building, beyond our traditional institutions of education.

**Declarations**

**Availability of data and materials**

The preprint version of this article (minus the experimental section) that was used in fiction condition is available in the supplementary materials, as well as the worksheets that were used in both conditions, and the exam questions that we used. The anonymized test results and survey responses are available in Supplemental Table 4.

**Competing interests**

The authors have no competing interests with this work.

**Funding**

This work was supported in part by NIH grants U54 CA217376, U2C CA233254, P01 CA91955, R01 CA170595, R01 CA185138 and R01 CA140657 as well as CDMRP Breast Cancer Research Program Award BC132057 and the Arizona Biomedical Research Commission grant ADHS18-198847. The findings, opinions and recommendations expressed here are those of the authors and not necessarily those of the universities where the research was performed or the National Institutes of Health.

**Authors' contributions**

CCM drafted the article and the supplemental materials. Both CCM and SS edited it. SS and CCM performed the statistical analyses.

**Acknowledgements**

We would like to thank Prof. Beckett Sterner who co-taught the evolution course with Prof Maley in the fall of 2020 and made this work possible. We would also like to thank Zachary Compton, Steven Messer and Katherine Weiss, who, along with Sareh Seyedi, were teaching assistants for the course, and ran the randomized tutorials for both the fact and fiction conditions of our experiment. We would also like to thank Athena Aktipis for her advice and Cristiano Parmeggiani and two anonymous reviewers for their thoughtful feedback.

**Authors' information**

Prof. Maley is an expert in the evolutionary biology of cancer and teaches the general evolution course at ASU. He is the director of the Arizona Cancer Evolution center which includes projects on applying life history theory to analyze patterns of cancer prevalence across species. He is also a lifelong fan of Lord of the Rings as well as fantasy world building and role playing.

Ms. Seyedi is a graduate student and PhD candidate in the Maley lab at the time of this writing. She is studying and doing her thesis on evolutionary strategy treatment for cancer disease.

**References:**

1. [Bixler A. Teaching Evolution with the Aid of Science Fiction. ambt. 2007 Aug;69(6):337–40.](http://paperpile.com/b/iKvXOj/Uez2d)

2. [Vrasidas C, Avraamidou L, Theodoridou K, Themistokleous S, Panaou P. Science Fiction in Education: case studies from classroom implementations. EMI Educ Media Int. 2015 Jul 3;52(3):201–15.](http://paperpile.com/b/iKvXOj/zxVuK)

3. [De Souza-Hart J. Creative ideas for biology podcasts: The immune system as an example. Am Biol Teach. 2011 Mar 1;73(3):171–5.](http://paperpile.com/b/iKvXOj/hYead)

4. [Tolkien JRR. The Letters of JRR Tolkien. Carpenter H, editor. Houghton Mifflin; 1981.](http://paperpile.com/b/iKvXOj/KYCPH)

5. [Carpenter H. J. R. R. Tolkien: A Biography. London : Allen and Unwin; 1977. 287 p.](http://paperpile.com/b/iKvXOj/0C8xp)

6. [Tolkien JRR. The Lord of the Rings: Being the Third Part of The Lord of the Rings. The Return of the King. Allen & Unwin; 1955.](http://paperpile.com/b/iKvXOj/kzuAI)

7. [Tolkien JRR. The Silmarillion. Tolkien C, editor. Houghton Mifflin; 1977.](http://paperpile.com/b/iKvXOj/4dlBo)

8. [Tolkien JRR, Tolkien C. The Peoples of Middle-Earth. Houghton Mifflin Harcourt; 1996.](http://paperpile.com/b/iKvXOj/dzsnG)

9. [Tolkien C, Tolkien JRR. Morgoth’s Ring. HarperCollins Publishers Limited; 2010. 500 p.](http://paperpile.com/b/iKvXOj/T0mJF)

10. [Tolkien JRR. The History of the Lord of the Rings. Tolkien C, editor. Houghton Mifflin; 2000. 1680 p.](http://paperpile.com/b/iKvXOj/wzulv)

11. [Tolkien JRR. Laws and Customs Among the Eldar. In: Tolkien C, editor. Morgoth’s Ring. HarperCollins Publishers Limited; 2010.](http://paperpile.com/b/iKvXOj/6m08n)

12. [Tolkien JRR. Athrabeth Finrod Ah Andreth. In: Tolkien C, editor. Morgoth’s Ring. HarperCollins Publishers Limited; 2010. p. Note 5.](http://paperpile.com/b/iKvXOj/akXrR)

13. [Lanner RM, Connor KF. Does bristlecone pine senesce? Exp Gerontol. 2001 Apr;36(4-6):675–85.](http://paperpile.com/b/iKvXOj/7l0LY)

14. [Tolkien JRR. The Lord of the Rings: The Fellowship of the Ring: being the first part of The Lord of the Rings. George Allen & Unwin; 1967.](http://paperpile.com/b/iKvXOj/qpMVL)

15. [Abegglen LM, Caulin AF, Chan A, Lee K, Robinson R, Campbell MS, et al. Potential Mechanisms for Cancer Resistance in Elephants and Comparative Cellular Response to DNA Damage in Humans. JAMA. 2015;1–11.](http://paperpile.com/b/iKvXOj/aP1qX)

16. [Plavcan JM. Sexual size dimorphism, canine dimorphism, and male-male competition in primates. Hum Nat. 2012;23(1):45–67.](http://paperpile.com/b/iKvXOj/pRpME)

17. [Frayer DW, Wolpoff MH. Sexual dimorphism. Annu Rev Anthropol. 1985;14(1):429–73.](http://paperpile.com/b/iKvXOj/jWXs2)

18. [Tolkien JRR. The Lord of the Rings: The Fellowship of the Ring. The Two Towers. The Return of the King. G. Allen and Unwin; 1971.](http://paperpile.com/b/iKvXOj/NBSXS)

19. [Tolkien JRR. Myths Transformed. In: Tolkien C, editor. Morgoth’s Ring. HarperCollins Publishers Limited; 2010.](http://paperpile.com/b/iKvXOj/jBFNT)

20. [Tolkien JRR. The Tale of Years of the Third Age. In: Tolkien C, editor. The Peoples of Middle-Earth. Houghton Mifflin Harcourt; 1996.](http://paperpile.com/b/iKvXOj/7rZtU)

21. [Tolkien JRR. The Hobbit, Or, There and Back Again. Ballantine Books; 1982. 304 p.](http://paperpile.com/b/iKvXOj/ZjFs2)

22. [Tolkien JRR. The Making of Appendix A: (IV) Durin’s Folk. In: Tolkien C, editor. The Peoples of Middle-Earth. Houghton Mifflin Harcourt; 1996.](http://paperpile.com/b/iKvXOj/VwS94)

23. [Tolkien JRR. Of Dwarves and Men. In: Tolkien C, editor. The Peoples of Middle-Earth. Houghton Mifflin Harcourt; 1996.](http://paperpile.com/b/iKvXOj/wVP1U)

24. [Novikov EA, Burda G. Ecological and evolutionary preconditions of extended longevity in subterranean rodents. Biology Bulletin Reviews. 2013 Jul 1;3(4):325–33.](http://paperpile.com/b/iKvXOj/49ZUA)

25. [Komdeur J. Helpers and Reproductive Behavior in Birds and Mammals [Internet]. Encyclopedia of Animal Behavior. 2010. p. 61–9. Available from:](http://paperpile.com/b/iKvXOj/9Tbz9) <http://dx.doi.org/10.1016/b978-0-08-045337-8.00193-5>

26. [Safran RJ, McGraw KJ. Plumage coloration, not length or symmetry of tail-streamers, is a sexually selected trait in North American barn swallows. Behav Ecol. 2004 May 1;15(3):455–61.](http://paperpile.com/b/iKvXOj/8plBj)

27. [Johnstone RA. Sexual selection, honest advertisement and the handicap principle: reviewing the evidence. Biol Rev Camb Philos Soc. 1995 Feb;70(1):1–65.](http://paperpile.com/b/iKvXOj/OXxs7)

28. [Zahavi A. Mate selection—a selection for a handicap. J Theor Biol. 1975;53(1):205–14.](http://paperpile.com/b/iKvXOj/nwKTw)

29. [Sinclair ARE, Mduma S, Brashares JS. Patterns of predation in a diverse predator–prey system. Nature. 2003 Sep;425(6955):288–90.](http://paperpile.com/b/iKvXOj/59ZM5)

30. [Wright NA, Steadman DW, Witt CC. Predictable evolution toward flightlessness in volant island birds. Proc Natl Acad Sci U S A. 2016 Apr 26;113(17):4765–70.](http://paperpile.com/b/iKvXOj/8Ew5S)

31. [Munshi-South J, Wilkinson GS. Bats and birds: Exceptional longevity despite high metabolic rates. Ageing Res Rev. 2010 Jan;9(1):12–9.](http://paperpile.com/b/iKvXOj/wOCJH)

32. [Fraser DF, Gilliam JF. Nonlethal Impacts of Predator Invasion: Facultative Suppression of Growth and Reproduction. Ecology. 1992 Jun 1;73(3):959–70.](http://paperpile.com/b/iKvXOj/riadi)

33. [Fournier D, Hellemans S, Hanus R, Roisin Y. Facultative asexual reproduction and genetic diversity of populations in the humivorous termite Cavitermes tuberosus. Proc Biol Sci [Internet]. 2016 Jun 15;283(1832). Available from:](http://paperpile.com/b/iKvXOj/hHC16) <http://dx.doi.org/10.1098/rspb.2016.0196>

34. [Boddy AM, Kokko H, Breden F, Wilkinson GS, Aktipis CA. Cancer susceptibility and reproductive trade-offs: a model of the evolution of cancer defences. Philos Trans R Soc Lond B Biol Sci [Internet]. 2015;370(1673). Available from:](http://paperpile.com/b/iKvXOj/RxWLu) <http://dx.doi.org/10.1098/rstb.2014.0220>

35. [De Queiroz K. Species concepts and species delimitation. Syst Biol. 2007 Dec;56(6):879–86.](http://paperpile.com/b/iKvXOj/QjkWw)

36. [Colin J-P. The Evolution of the Peoples of Middle-Earth: A Phylogenetic Approach to Humanoids in Tolkien. In: Lehoucq R, Mangin L, Steyer J-S, editors. The Science of Middle-Earth. New York: Pegasus Books, Ltd.; 2021. p. 263–74.](http://paperpile.com/b/iKvXOj/V1GI)

37. [Crowe A, Dirks C, Wenderoth MP. Biology in bloom: implementing Bloom’s Taxonomy to enhance student learning in biology. CBE Life Sci Educ. 2008 Winter;7(4):368–81.](http://paperpile.com/b/iKvXOj/lNKE3)

1. Though Arwen herself carries some human genes through her father’s side. [↑](#footnote-ref-0)
