# Supplementary material for: The life history theory of the Lord of the Rings: a randomized controlled trial of using fact versus fiction to teach life history theory
Source: Evolution (N Y). 2022 Feb 16;15(1):2. doi: 10.1186/s12052-022-00160-8 (PMC8850221; doi:10.1186/s12052-022-00160-8)
Supplement: Supplementary file 2 — Additional file 2: Text S2. A six question worksheet for the fiction article. [file 12052_2022_160_MOESM2_ESM.docx]

**Question 1**

Place elves, dwarves, orcs, humans and hobbits as points on a reproduction-survival-growth trade-off triangle diagram.


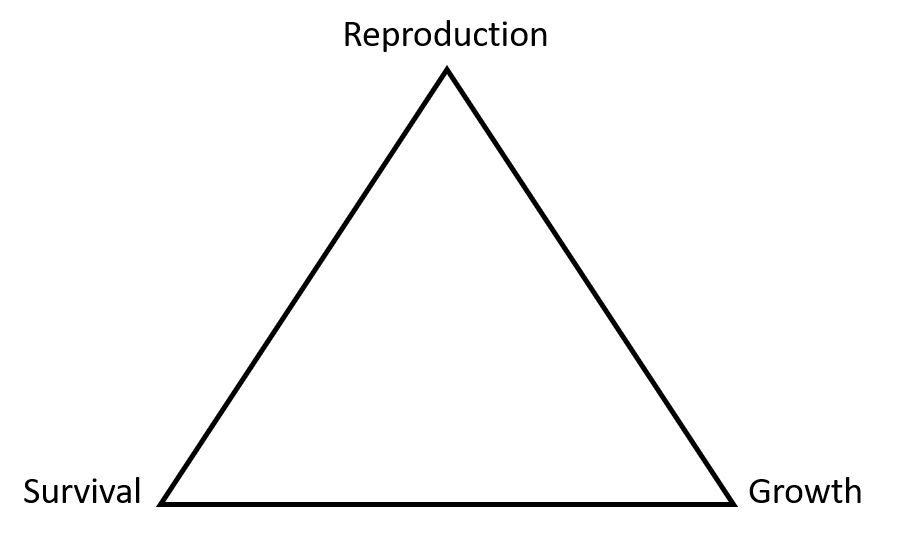


**Question 2**

Why can we infer that violent death was extremely rare during the evolution of elves? Explain your reasoning.

**Question 3**

If orcs had won their wars, leading to the extinction of their traditional enemies (humans, elves, and dwarves) in Middle-Earth, so that the likelihood of their violent death dramatically decreased, what would you predict would happen to their population size over time, and how would you predict their lifespan and fecundity would evolve in response to their new ecology? Explain your reasoning.

**Question 4**

Identify two species you know that have a slow life history (that were not mentioned in class or the texts you read about life history strategies). Provide what you know about their lifespan, reproductive output and growth.

**Question 5**

Identify **two species** you know that have a **fast life history** (that were not mentioned in class or the texts you read about life history strategies). Provide what you know about their lifespan, reproductive output and growth.

**Question 6**

Choose a non-human species from a fictional source (book, movie, etc.):

- 1. Where does it fall on the slow to fast life history continuum? What is your evidence for that?
  2. From what you know of its life history strategy, what other aspects of its biology can you infer?
