# Supplementary material for: The life history theory of the Lord of the Rings: a randomized controlled trial of using fact versus fiction to teach life history theory
Source: Evolution (N Y). 2022 Feb 16;15(1):2. doi: 10.1186/s12052-022-00160-8 (PMC8850221; doi:10.1186/s12052-022-00160-8)
Supplement: Supplementary file 4 — Additional file 4: Text S4. Eight multiple choice questions used for our exam. [file 12052_2022_160_MOESM4_ESM.docx]

EXAM Questions:

1. Calculate the growth rate (R = the average number of offspring produced by an individual) of the species described by the life history table below. Remember that lx is the chance of surviving to age x, and mx is the average number of offspring produced at age x.

| Age | Survival Probability ( lx) | Fecundity (mx) | lxmx |
| --- | --- | --- | --- |
| 0 | 0.90 | 0 |  |
| 1 | 0.89 | 0 |  |
| 2 | 0.50 | 0 |  |
| 3 | 0.25 | 4 |  |
| 4 | 0.20 | 10 |  |
| 5 | 0.1 | 20 |  |

R = ?

1. Consider the life history table of a pest below. Imagine that a farmer introduces a new predator to the environment that eats adult pests. What would you predict would happen to the values in the cells labeled A and B in the life history table?

| Age | Survival Probability ( lx) | Fecundity (mx) |
| --- | --- | --- |
| 0 | 0.90 | 0 |
| 1 | 0.89 | **A.** 1 |
| 2 | 0.50 | 1 |
| 3 | 0.25 | 4 |
| 4 | **B**. 0.20 | 10 |
| 5 | 0.1 | 20 |

Circle the correct answer

A (fecundity at age 1)

**would likely decrease would likely increase would likely stay the same**

B (survival probability at age 4)

**would likely decrease would likely increase would likely stay the same**

1. If there is a creature that has the ability to live for 300 years but is extremely prone to dying by disease, what other life history characteristics would you expect to see in that species? Select all that apply
2. Late reproduction
3. A lot of offspring
4. Small body size
5. Few offspring
6. Large body size
7. Early reproduction
8. In a forest, shade intolerant weeds can co-exist with tall woody trees because
9. Weeds are typically resistant to fungi and insects
10. Weeds can outcompete woody trees
11. Weeds invest in dispersal to find areas of recent disturbances
12. Weeds produce more seeds than woody trees
13. As an intrepid explorer, you discover a new species of mammal that has the following life history characteristics:

- It takes a long time to reach maturity
- It has a slow reproduction rate
- It has a long lifespan

What other things would you predict from life history theory?

1. It will produce offspring that are large in size
2. It will produce only one offspring at a time
3. It will produce a large number of offspring
4. There are high levels of external mortality
5. What are the main trade-offs identified in life history theory? (You may select multiple answers)
6. Stress tolerance
7. Survival
8. Reproduction
9. Dispersal
10. Growth
11. Mate competition
12. If older individuals of a species have higher social status and get more mating opportunities, what would you predict about the following life history characters?

(Note: The same prediction may apply to multiple characters, and some predictions may not apply to any character.)

Somatic maintenance (circle the correct answer)

**would be high would be moderate would be low**

Cancer defense mechanisms (circle the correct answer)

**would be high would be moderate would be low**

Number of offspring (circle the correct answer)

**would be high would be moderate would be low**

1. If the predator of a species goes extinct, what would you predict would happen to the prey species and why?

(Note: The same explanation may apply to multiple predictions, and some explanations do not apply to any prediction.)

Group of answer choices

**Its population size would stabilize after a period of growth**

A. Because the primary selective pressure is now competition for limited resources.

B. Because the species' primary selective pressure is now external mortality.

C. Because whichever lineage produces the most offspring the fastest will sweep through the population by natural selection.

D. Because the species reaches the carrying capacity of its environment.

E. Because the species needs to reproduce quickly before they die of some other cause.

F. No, this is not a prediction of life history theory under these conditions.

**Its body size would increase**

A. Because the primary selective pressure is now competition for limited resources.

B. Because the species' primary selective pressure is now external mortality.

C. Because whichever lineage produces the most offspring the fastest will sweep through the population by natural selection.

D. Because the species reaches the carrying capacity of its environment.

E. Because the species needs to reproduce quickly before they die of some other cause.

F. No, this is not a prediction of life history theory under these conditions.

**Its parental investment in offspring would increase**

A. Because the primary selective pressure is now competition for limited resources.

B. Because the species' primary selective pressure is now external mortality.

C. Because whichever lineage produces the most offspring the fastest will sweep through the population by natural selection.

D. Because the species reaches the carrying capacity of its environment.

E. Because the species needs to reproduce quickly before they die of some other cause.

F. No, this is not a prediction of life history theory under these conditions.

**They would produce more offspring**

A. Because the primary selective pressure is now competition for limited resources.

B. Because the species' primary selective pressure is now external mortality.

C. Because whichever lineage produces the most offspring the fastest will sweep through the population by natural selection.

D. Because the species reaches the carrying capacity of its environment.

E. Because the species needs to reproduce quickly before they die of some other cause.

F. No, this is not a prediction of life history theory under these conditions.

**They would grow rapidly**

A. Because the primary selective pressure is now competition for limited resources.

B. Because the species' primary selective pressure is now external mortality.

C. Because whichever lineage produces the most offspring the fastest will sweep through the population by natural selection.

D. Because the species reaches the carrying capacity of its environment.

E. Because the species needs to reproduce quickly before they die of some other cause.

F. No, this is not a prediction of life history theory under these conditions.
